# The effects of aerobic and resistance exercise on blood pressure in uncomplicated and at risk pregnancies: A systematic review and meta-analysis

**DOI:** 10.1177/17455057231183573

**Published:** 2023-07-16

**Authors:** Courtney Giles, Rich Johnston, Jade Kubler, Jemima Spathis, Kassia Beetham

**Affiliations:** 1 School of Behavioural and Health Sciences, Australian Catholic University, Brisbane, QLD, Australia; 2 Sports Performance, Recovery, Injury and New Technologies Research Centre, Australian Catholic University, Brisbane, QLD, Australia; 3 Carnegie Applied Rugby Research Centre (CARR), Carnegie School of Sport, Leeds Beckett University, Leeds, UK; 4 Mater Research Institute, The University of Queensland, South Brisbane, QLD, Australia

**Keywords:** blood pressure, exercise, gestational hypertension, haemodynamics, pregnancy

## Abstract

**Background:**

Regular exercise performed during pregnancy has been shown to reduce the risk of developing perinatal gestational hypertensive conditions. Further evidence on the exact parameters of exercise needed to explain these beneficial responses is required, within both uncomplicated and at-risk pregnancies.

**Objective:**

The aim of this systematic review and meta-analysis was to investigate the effects of aerobic and resistance exercise on blood pressure during pregnancy.

**Design:**

Systematic review and meta-analysis.

**Data Sources and Methods:**

An online search of six search engines was conducted up to February 2023. Randomized controlled trials, quasi-experimental, cohort, and longitudinal studies were included. Studies included an acute exercise bout or intervention of land-based aerobic and/or resistance exercise during any trimester in uncomplicated and at-risk pregnancies. Outcomes included mean arterial pressure (MAP), or systolic blood pressure (SBP) and diastolic blood pressure (DBP).

**Results:**

Following the removal of duplicates, 1538 articles were screened with 59 studies meeting the inclusion criteria for the review (randomized controlled trials (RCTs) n = 34, clinical trials n = 19, cohort n = 5 and cross-sectional n = 2), and 21 studies included in the meta-analysis. A random effects model was used with mean difference calculated in mmHg. Overall, there were no statistically significant effects of exercise on resting blood pressure (BP) outcomes in pregnant women with normal blood pressure compared to control/usual care populations following intervention (SBP mean diff -1.54 mmHg (favours intervention), p = 0.38; DBP mean diff -2.25 mmHg (favours intervention), p = 0.1; MAP mean diff -1.75 mmHg (favours intervention), p = 0.31). In at-risk pregnant women, both aerobic and combination exercise significantly reduced BP outcomes compared to control (SBP mean diff -3.91 mmHg, p < 0.01; DBP mean diff -2.9 mmHg, p = 0.01; MAP mean diff -2.38 mmHg, p = 0.01). Twenty-seven studies reported an acute increase in SBP and DBP during aerobic exercise, with no difference found between uncomplicated and at-risk pregnancies.

**Conclusions:**

Compared to usual care, aerobic and/or resistance exercise performed throughout uncomplicated pregnancy had no influence on blood pressure. Pregnant women with no diagnosed complications should be encouraged to exercise regularly due to the multitude of known benefits. In women who are at risk of, or diagnosed, with gestational hypertensive conditions during pregnancy, moderate to vigorous exercise during pregnancy improves blood pressure outcomes. Higher risk pregnancies may reduce their risk of future cardiovascular complications through regular exercise training during pregnancy.

**Registration:**

CRD42020159998.

## Introduction

Pregnancy is a period characterized by significant physiological adaptations, particularly within the cardiovascular system.^1^ Maternal haemodynamic alterations within the cardiovascular system are evident from the first few weeks of gestation.^2,3^ These rapid changes are necessary to ensure sufficient uteroplacental blood flow to transfer oxygen and nutrients from the mother to the foetus, to optimize foetal development.^4,5^ An increase in heart rate (HR), cardiac output (CO), stroke volume (SV), and plasma volume are observed in healthy pregnancies and associated with a concomitant fall in total vascular resistance and systemic vascular tone.^6,7^ Maladaptive changes to these maternal haemodynamic processes can occur during gestation, increasing the risk of gestational hypertensive conditions.^2,4^

Pre-eclampsia (PE) and gestational hypertension (GHTN) are pregnancy-specific disorders that pose a significant risk to pregnant women, with the World Health Organization (WHO) recognizing these conditions among the leading causes of maternal and foetal morbidity and mortality worldwide, along with haemorrhage and sepsis.^8–10^ The exact cause of GHTN and PE are not well established; however, it has been identified that hypertensive conditions that present prior to 20 weeks of gestation (chronic HTN, GHTN) often advance to PE.^9,11–13^ The vascular dysfunction that is associated with gestational hypertensive conditions is considered systemic and persistent resulting in a significantly increased risk of future cardiovascular disease (CVD).^5,9,14^ Infants born following pre-eclamptic pregnancy have also been shown to be at an increased risk for childhood obesity and CVD later in life.^12,15^ Other clinical conditions such as gestational diabetes mellitus (GDM) and overweight/obesity significantly increase the risk of developing hypertensive conditions in pregnancy.^16^

There is convincing evidence that both acute and long-term aerobic, and resistance, exercise, from light to vigorous intensity, lowers resting blood pressure (BP) in both hypertensive and normotensive non-pregnant populations.^17,18,19^ Regular physical activity has been shown to positively enhance metabolic and musculoskeletal changes associated with pregnancy; however, the mechanisms of prenatal exercise on blood pressure are not yet well understood.^14,20^ Two recent systematic reviews looked at the effects of prenatal exercise on measures of cardiovascular health including blood pressure, and found that resting blood pressure was reduced following prenatal exercise interventions.^21^ Furthermore, the risk of developing major clinical conditions such as GHTN, PE, and GDM is significantly reduced in women who engaged in regular prenatal exercise.^22^ There is, however, a lack of understanding surrounding the effects of different types and intensities of prenatal exercise on maternal blood pressure,^14^ as well as whether uncomplicated and at-risk populations respond differently to prenatal exercise. Further evidence on the exact parameters of exercise needed to elucidate these beneficial responses is required.

The primary aim of this systematic review and meta-analysis is to determine the effects of acute and long-term aerobic exercise, resistance exercise and a combination of both, on blood pressure outcomes in uncomplicated and at-risk pregnant populations. It is hypothesized that acute bouts of aerobic exercise will result in post exercise hypotensive responses, and that long-term aerobic exercise during pregnancy will reduce blood pressure and help prevent the onset of gestational hypertensive disorders, particularly within populations who are at increased risk of these conditions.

## Methods

This systematic review and meta-analysis was conducted in accordance with the Preferred Reporting Items for Systematic Reviews and Meta-Analyses (PRISMA) guidelines.^23^ The review was registered with PROSPERO (International Prospective Register for Systematic Reviews) under the registration number CRD42020159998.^24^

### Search strategy

Six online search engines (CINAHL, Cochrane, Embase, Medline, PubMed, Web of Science) were used to search databases up to February 2023. Standardized search terms were established with pregnant women as the population, aerobic or resistance exercise as the intervention, and MAP or BP as the primary outcome measures. All synonymous terms that may be used to describe the population, intervention and outcome were included. Medical Subject headings (MeSH), truncation, and Boolean operators were used to ensure that all relevant articles were found in the database searches. Filters were applied to ensure searches were limited to studies on humans and reported in English. The reference lists of included articles were screened to ensure that any relevant studies missed in the database searches could be included in the review. The complete search strategy for each search engine can be viewed in Supplementary File 1.

### Eligibility criteria

The types of studies eligible to be included in this review were randomized controlled trials (RCTs), quasi-experimental, cohort, longitudinal, case-control, or non-randomized intervention studies. To be eligible for inclusion in the review, studies needed to be peer-reviewed articles including (1) pregnant women completing either an acute bout or an intervention of land-based aerobic or resistance exercise during any trimester, and (2) maternal MAP or SBP/DBP reported as either a primary or secondary outcome measure. To be included in the meta-analysis, studies required the above listed criteria, along with a comparator/control group treated with standard prenatal care. Studies that reported on water-based activities were excluded due to the thermal effects of both warm and cold-water immersion on the cardiovascular system.^25^ Only primary studies were included in the review, to ensure that data from these studies were only taken into consideration once. Both uncomplicated and at-risk pregnant populations were included.

### Definitions

The methodologies of the articles were reviewed in detail to determine whether the exercise intervention included in the study met the criteria for land-based aerobic, resistance, or combination exercise. The American College of Sports Medicine (ACSM) defines aerobic exercise as any activity that uses large muscle groups, is rhythmic in nature, and can be maintained continuously, while resistance exercise involves exercising muscles against an external load or resistance in order to improve muscular fitness.^26^ Studies including aerobic or resistance land-based exercise at any intensity were included.^27^ Acute exercise is defined as a single bout of exercise following which researchers observed any changes between pre- and post-exercise outcome measures. Exercise interventions are defined as repeated bouts of exercise across a period of time (in this case ⩾ 3 weeks) following which researchers observed any changes between pre- and post-intervention outcome measures. The intensity of exercise was determined based on percentage of heart rate max (%HRmax) and rating of perceived exertion (RPE) on the Borg Scale and rated as light (40%–55% HRmax, RPE: 8-10), moderate (55%–70% HRmax, RPE: 11-13), vigorous (70 < 90% HRmax, RPE 14-16), or high (⩾ 90% HRmax, RPE: > 17).^27^ In this review, an at risk pregnancy is defined as one with diagnosed conditions that increase the pregnant woman’s risk of developing gestational hypertensive conditions, including but not limited to: GDM, overweight/obesity, chronic hypertension, and/or previous pre-eclampsia.^16^ Uncomplicated pregnancies are defined as those with no pre-existing medical comorbidities (e.g. HTN, type 2 diabetes) and no pre-existing or new-onset obstetric complications (e.g. PE, GDM).^28^

### Assessment of risk of bias

The Cochrane Risk of Bias for Randomized Controlled Trials tool was used to assess the risk of bias in the RCTs and randomized clinical trials (Supplementary File 2).^29^ This assessment tool allowed the authors to assess the bias in each study as low, high, or unclear across six domains including: selection bias, reporting bias, detection bias, performance bias, attrition bias, and other bias. Based on the scores in each domain an overall risk of bias score was generated as low, unclear, or high risk.

The Newcastle-Ottawa Scale was used to assess the cohort and case control studies. Eight questions are used to assess quality based on comparability, selection, outcomes for the cohort studies, and exposure for the case-control studies.^30^ A total of the scores out of nine is then calculated to provide an overall quality assessment. Three reviewers (C.G., J.S., and J.K.) conducted the bias assessments separately and discussed any discrepancies to come to a consensus.

The Revised Cochrane risk of bias tool for randomized trials (RoB 2) with additional considerations for crossover trials was used to assess the bias present in the crossover trial.^31^ This tool assesses risk of bias across five domains including (1) randomization process, (2) deviations from intended intervention, (3) missing outcome data, (4) measurement of the outcome, and (5) reporting of results. Each domain is judged as low, some concern, or high risk, and then an overall risk of bias is determined. In order to determine the risk of bias in non-randomized single-arm clinical trials, five questions were selected from the Newcastle-Ottawa scale, which has been previously described as a method of assessing these studies.^32^

### Data collection process

The results from the database searches were exported to EndNote X9 for the screening process. Duplicates were removed, and the titles and abstracts were screened by C.G. . The full texts of the included articles were retrieved for screening and reviewed in full by CG and JK. The data extracted from the studies was screened separately by two reviewers (C.G. & J.K.) to ensure the studies met the eligibility criteria. A third reviewer (J.S.) provided an evaluation if there were any discrepancies. The following information was extracted from the studies: study design, sample size, year and location, participant characteristics, intervention and control conditions, SBP, DBP and MAP (calculated) as well as information used to conduct the risk of bias assessment (Supplementary File 3).

### Statistical analysis

The primary outcomes in this study were the impact of exercise during pregnancy on SBP, DBP and MAP. Meta-analyses were conducted for all instances in which two or more studies reported data on comparable outcomes, interventions, participants and comparators as recommended by Ioannidis and Rothstein.^33^ Only two studies reported MAP as an outcome measure; therefore, the SBP and DBP reported in each of the studies was used to calculate the MAP for the control and exercising groups using the equation^34^

$$\frac{SBP+\left(2DBP\right)}{3}=MAP$$

The sample standard deviation for each of the calculated MAPs was found using the standard variances for each measure. The following equations were used, where SD1 is the SBP SD and SD2 is the DBP SD

$$SD1^{2}=SV1$$

$$SD2^{2}=SV2$$

$$\frac{SV1+\left(2SV2\right)}{3}=MAP\;SV$$

$$\sqrt{MAP\;SV}=MAP\;SD$$

The software Review Manager 5 (RevMan V5, The Cochrane Collaboration) was utilized to run random effects meta-analysis using the DerSimonian and Laird method to estimate between-study variance. Meta-analyses were conducted separately for each outcome – SBP, DBP, and MAP. Subgroup analysis was performed to determine any effect of exercise type on outcome measures. As all resting blood pressure measures were recorded in mmHg, unstandardised mean differences were calculated for these continuous outcomes within each study. Standard variance was used to calculate the standard deviation when these were not reported by studies. Heterogeneity between studies was then assessed based on the *I*^2^ value for each analysis, with an *I*^2^ value between 30% and 60% considered moderate, and any value higher than 60% considered substantial heterogeneity.^35^ Leave-one-out analysis was performed to determine the effect of each study on the heterogeneity.

## Results

### Study selection

**Figure 1.**
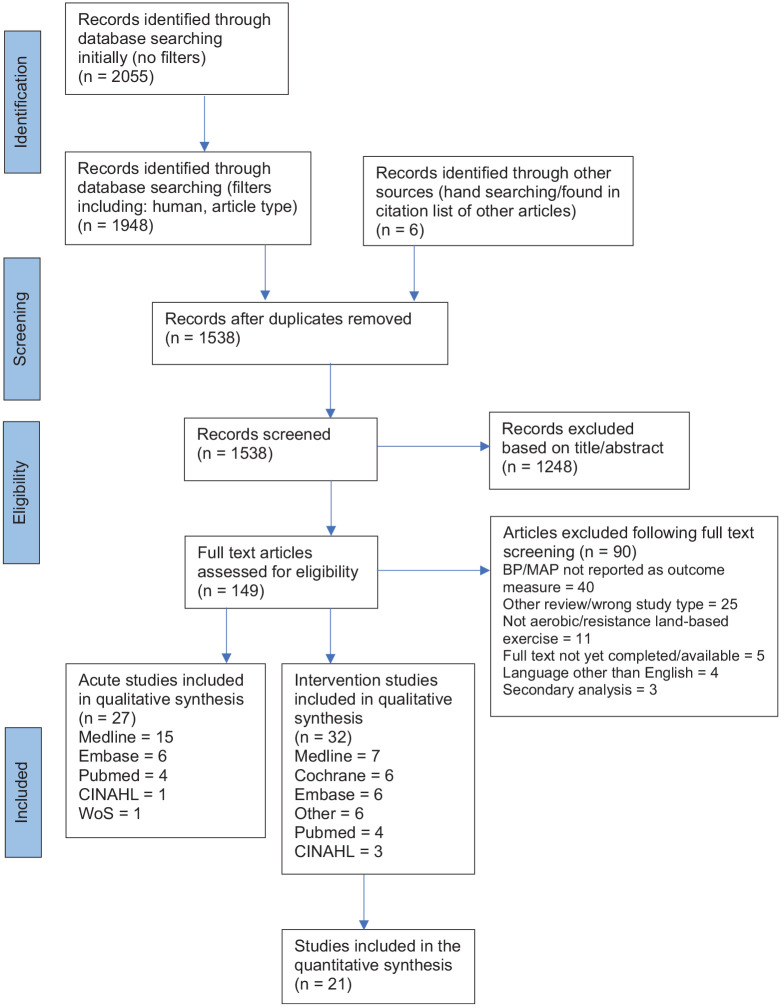
PRISMA flow chart.

The screening process of the studies can be viewed in Figure 1. In the initial search, 2055 articles were identified (CINAHL: 216, Cochrane: 1072, Embase: 107, Medline: 441, PubMed: 115, Web of Science: 104). Filters were applied, duplicates were removed and the titles and abstracts were screened for eligibility. Full texts were screened, and 59 articles were found to be eligible for the review. There were 32 exercise intervention studies and 27 acute exercise studies. Four of the intervention studies also reported acute responses to exercise. The types of studies included were RCTs (n = 33), clinical trials (n = 19), cohort (n = 5), and cross-sectional (n = 2). Eight intervention studies were included in the review that discussed BP, however did not report either pre or post SBP, DBP, or MAP values, or did not include a control/comparator group.^36–43^ These studies were not included in the meta-analysis, along with one study which failed to report SD for SBP or DBP,^44^ leaving 21 intervention studies in the statistical analysis. In the 27 acute studies, the gestational age at the time of the study, modality of exercise, and whether the final outcome measure was measured at rest or during exercise varied considerably; therefore, the acute studies were not included in the meta-analysis and are narratively presented. Six studies were excluded as they included water-based activities rather than land-based aerobic or resistance exercise.^45–50^ These aquatic-based studies did not fit within the inclusion criteria for this review; however, this is an important area of research given that swimming is a popular, low-impact exercise during pregnancy.

### Risk of bias

The risk of bias can be viewed in Supplementary file 2 (Tables S1–S5). Overall, the risk of bias in the RCTs and randomized clinical trials was low, with 27 (71%) studies assessed as low risk,^20,34,36–39,41,42,51–69^ 10 (27%) studies classified as unclear^44,69–76^ and one (2%) study considered high risk.^77^ The cohort^78–84^ and case control studies^85–90^ were all classified as low (71%) to moderate (29%) risk of bias (Tables S2 and S3), as were the two crossover studies (Table S4).^91,92^ One (15%) of the single-arm clinical trials was found to have an unclear risk of bias,^93^ while the other six (85%) studies were deemed to be low risk (Table S5).^40,43,94–97^

### Characteristics of acute and long-term exercise interventions

The characteristics of the participants included in the intervention and acute studies can be found in Tables S6 and S7, respectively (Supplementary File 3). The designs of the exercise interventions and acute studies can be viewed in Table S8 (Supplementary File 3) and Table S9 (Supplementary File 3) respectively.

### Meta-analysis

#### Pooled results–uncomplicated pregnancies

Data were pooled from 13 studies to assess the effect of a long-term exercise intervention on SBP, and from 12 studies to assess DBP and MAP in uncomplicated pregnancies. There was no significant effect of exercise compared to control on the change in SBP (mean difference [95% CL] -1.54 mmHg [-5, 1.93], p = 0.38, Tau^2^ = 37.34, Chi^2^ = 1792.51, df = 12, I^2^ = 99%), DBP (mean difference [95% CL] –2.25 mmHg [-4.96, 0.45], p = 0.1, Tau^2^ = 20.78, Chi^2^ = 774.07, df = 11, I^2^ = 99%), or MAP (mean difference [95% CL] -1.75 mmHg [-5.13–1.63], p = 0.31, Tau^2^ = 31.75, Chi^2^ = 1000.16, df = 11, I^2^ = 99%) when aerobic, resistance, and combination exercise studies were pooled.

#### Pooled results – at risk population

Within the 10 at-risk studies the pooled data showed a significant effect of exercise on SBP (mean difference [95% CL] –3.91 mmHg, [-6.74, -1.08], p = < 0.01, Tau^2^ = 16.52, Chi^2^ = 160.29, df = 9, I^2^ = 94%), DBP (mean difference [95% CL] -2.9 mmHg [-5.11, -0.68], p = 0.01, Tau^2^ = 10.47, Chi^2^ = 244.97, df = 9, I^2^ = 96%), and MAP (mean difference [95% CL] -2.38 mmHg [-4.27, -0.48], p = 0.01, Tau^2^ = 6.61, Chi^2^ = 255.06, df = 8, I^2^ = 97%) compared to the control group.

### Aerobic exercise interventions

#### Uncomplicated pregnancies

**Figure 2.**
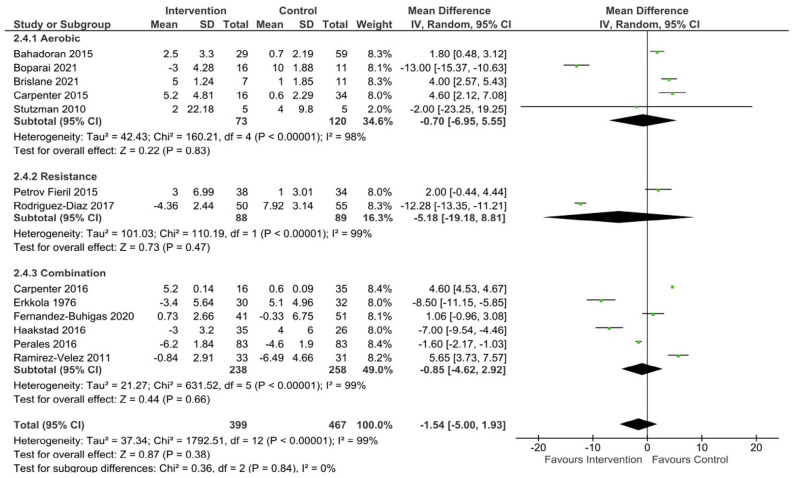
SBP changes following exercise in uncomplicated pregnancies.

**Figure 3.**
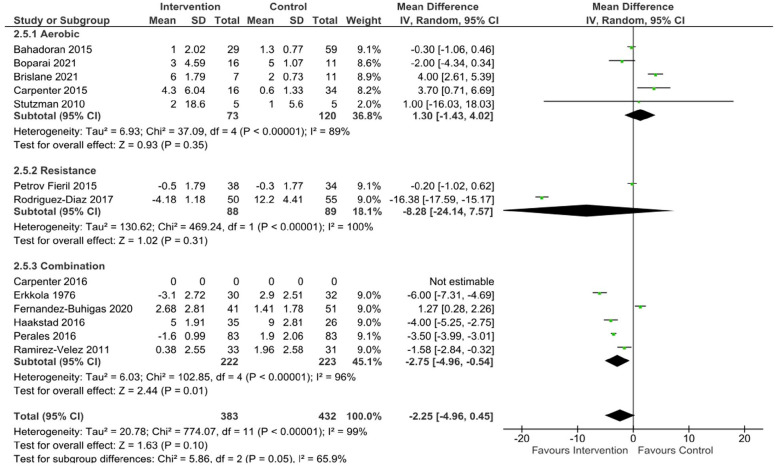
DBP changes following exercise in uncomplicated pregnancies.

**Figure 4.**
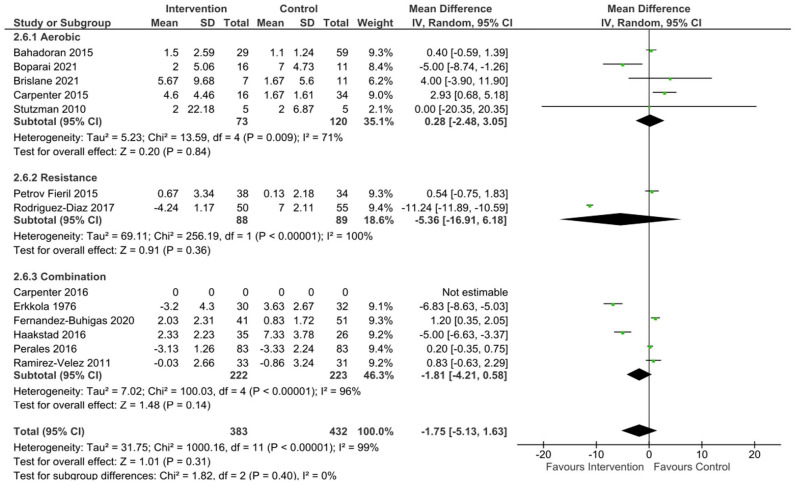
MAP changes following exercise in uncomplicated pregnancies.

Six studies included aerobic exercise interventions within uncomplicated pregnant populations,^39,53,66,70,72,74^ with only one study not meeting the inclusion criteria for the meta-analysis.^39^ The meta-analysis showed no significant difference in SBP (mean difference [95% CL] = -0.70 mmHg [-6.95, 5.55], p = 0.83, Tau^2^ = 42.43, Chi^2^ = 160.21, df = 4, I^2^ = 98%) (Figure 2), DBP (mean difference [95% CL] = 1.30 mmHg [-1.43, 4.02], p = 0.35, Tau^2^ = 6.93, Chi2 = 37.09, df = 4, I^2^ = 89%)(Figure 3), and MAP (mean difference [95% CL] = 0.28 mmHg [-2.48, 3.05], p = 0.84, Tau^2^ = 5.23, Chi^2^ = 13.59, df = 4, I^2^ = 71%) (Figure 4) between healthy exercising and control groups following aerobic exercise interventions. The leave-one-out analysis showed a large drop in heterogeneity when one study^53^ was excluded from the SBP data (mean difference [95% CL] = 3.26 mmHg [1.62, 4.89], p = 0.08, Tau^2^ = 1.35, Chi^2^ = 6.84, df = 3, I^2^ = 56%) and the MAP data (mean difference [95% CL] = 1.45 mmHg [-0.38, 3.29], p = 0.12, Tau^2^ = 1.21, Chi^2^ = 4.68, df = 3, I^2^ = 36%).

#### At risk populations

**Figure 5.**
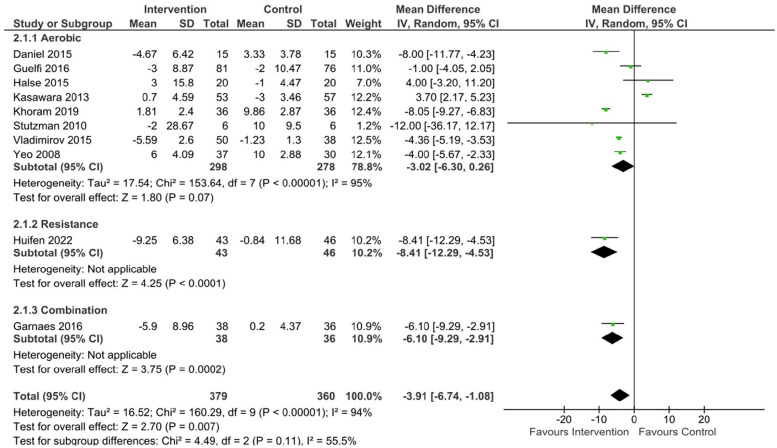
SBP changes following exercise in at risk populations.

**Figure 6.**
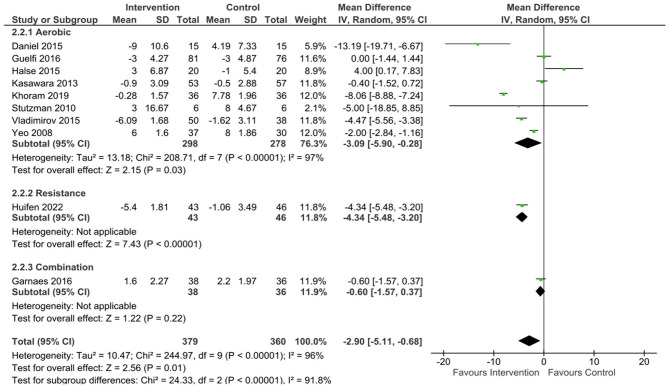
DBP changes following exercise in at risk populations.

**Figure 7.**
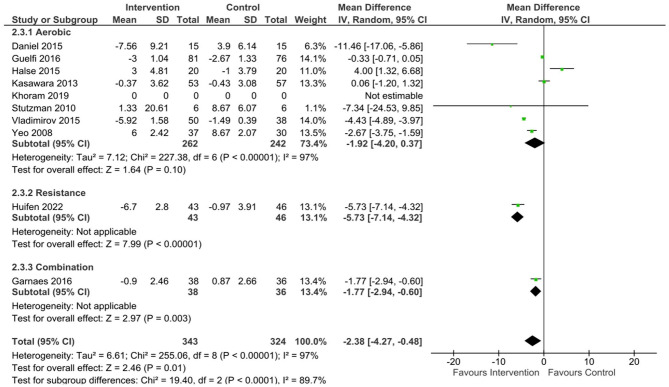
MAP changes following exercise in at risk populations.

Ten studies included at risk populations performing aerobic exercise interventions, seven of which were included in the statistical analysis.^54,57,58,60,66–68^ The clinical conditions included: overweight/obesity (body mass index (BMI) > 25 kg/m^2^),^66,98^ GDM or history of GDM,^54,57,58^ anaemia,^67^ or high risk of GHTN/PE due to chronic or mild HTN, previous GHTN/PE or family history of HTN/PE.^41,44,60,68^ Following aerobic exercise, a near significant difference was found for SBP (mean difference [95% CL]= -3.02 mmHg [-6.3, 0.26], p = 0.07, Tau^2^ = 17.54, Chi^2^ = 153.64, df = 7, I^2^ = 95%) (Figure 5) and MAP (mean difference [95% CL] = -1.92 mmHg [-4.2, 0.37], p = 0.1, Tau^2^ = 7.12, Chi^2^ = 227.38, df = 6, I^2^ = 97%) (Figure 7) between exercising and control groups. A statistically significant reduction in DBP (mean difference [95% CL] = -3.09 mmHg [-5.9, -0.28], p = 0.03, Tau^2^ = 13.18, Chi^2^ = 208.71, df = 7, I^2^ = 97%) (Figure 6) was found in the at risk population following aerobic exercise compared to control.

The three studies excluded from the analysis did not report baseline and post-intervention SBP and DBP; therefore, the mean change in these measures could not be calculated to be used in the analysis.^41,44,98^ No significant differences in SBP or DBP were discussed by the studies. Long-term changes in SBP and DBP were not reported by Khoram et al.^41^; however, acute responses to exercise were discussed. There was a significantly lower incidence of PE and GHTN in the exercising group compared to control (p = < 0.05). Results from Yeo et al.^44^ showed no significant difference in BP between groups; however, both SBP and DBP reduced in the exercising group and increased in the control group with a near significant difference in DBP found with a reduction of 3.5 mmHg in the exercising group and an increase of 1.1 mmHg in the control group (p = 0.05). Changes in BP from baseline were not reported by Senevirante et al.^98^; however, there were no significant differences in mean SBP (p = 0.25) or DBP (p = 0.68) between exercising and control groups.

### Resistance exercise interventions

#### Uncomplicated pregnancies

Four studies included in the review involved an intervention of supervised low to moderate intensity strength training.^40,43,63,64^ Two of these did not include a comparator/control group leaving only two studies eligible for inclusion in the analysis.^63,64^ No significant differences were seen between groups in the two studies included in the meta-analysis for SBP (mean difference [95% CL] = -5.18 mmHg [-19.18, 8.81], p = 0.47, Tau^2^ = 101.03, Chi^2^ = 110.19, df = 1, I^2^ = 99%) (Figure 2), DBP (mean difference [95% CL] = -8.28 mmHg [-24.14, 7.57], p = 0.31, Tau^2^ = 130.62, Chi^2^ = 469.24, df = 1, I^2^ = 100%) (Figure 3), or MAP (mean difference [95% CL] = -5.36 mmHg [-16.91, 6.18], p = 0.36, Tau^2^ = 69.11, Chi^2^ = 256.19, df = 1, I^2^ = 100%) (Figure 4). The results from the two studies not included in the meta-analysis showed no significant changes in SBP (113.5 ± 8.4 mmHg to 113.9 ± 10 mmHg;^43^ 108 ± 13.5mmHg to 113.1 ± 9.12)^40^ or DBP (71.9 ± 6.8 mmHg to 73.3 ± 7.1 mmHg;^43^ 66.8 ± 10.1 mmHg to 70.6 ± 10.4 mmHg)^40^ following the interventions.

#### At risk populations

Two studies included at-risk populations performing resistance training^42,59^; however, only one of these reported baseline and postintervention SBP and DBP^59^; therefore, no subgroup analysis could be run, as at least two studies are required.^33^ One study reported a significant decrease in SBP (Pre: 121.37 ± 15.83 mmHg, Post: 112.12 ± 13.87 mmHg; p = < 0.001) and DBP (Pre: 75.63 ± 8.96 mmHg, Post: 70.23 ± 7.38 mmHg; p < 0.001) in the intervention group compared to control (SBP Pre: 119.8 ± 17.47 mmHg, Post: 118.96 ± 17.38; p = 0.12; DBP Pre: 75.65 ± 10.86 mmHg, Post: 74.59 ± 10.94mmHg; p = 0.15).^59^ Arterial BP was reported as a secondary outcome measure in the other RCT with no significant differences found for either SBP or DBP following resistance training.^42^

### Combination interventions

#### Uncomplicated pregnancies

No significant differences were found in SBP (mean difference [95% CL] = -0.85 mmHg [-4.62, 2.92], p = 0.66, Tau^2^ = 21.27, Chi^2^ = 631.52, df = 5, I^2^ = 99%) (Figure 2) or MAP (mean difference [95% CL] = -1.81 mmHg [-4.21, 0.58], p = 0.14, Tau^2^ = 7.02, Chi^2^ = 100.03, df = 4, I^2^ = 96%) (Figure 4). A small but statistically significant reduction in DBP was found following an intervention of combined aerobic and resistance exercise compared to control (mean difference [95% CL] = -2.75 mmHg [-4.96, -0.54], p = 0.01, Tau^2^ = 6.03, Chi^2^ = 102.85, df = 4, I^2^ = 96%) (Figure 3). Four studies were not included in the analysis as they did not report on the change in BP from baseline.^36–38,65^ Three studies did not report baseline BP, however, found no significant differences between groups for SBP (p = > 0.05;^37^ p = 0.25;^36^ p = 0.49)^38^ or DBP (p = > 0.05;^37^ p = 0.29;^36^ p = 0.74)^38^ postintervention. One study found no differences in SBP or DBP across the three trimesters between intervention and control in a study of 72 women.^65^

#### At-risk populations

Only one study reported changes in BP following an intervention of combination exercise in an at risk population and found the mean SBP of the exercising group was significantly lower than the control group following intervention, with a mean reduction of 7.7 mmHg (95% CI −13.23, −2.22; p < 0.001) and no significant difference in DBP or MAP between groups.^56^

### Acute aerobic exercise

#### Uncomplicated pregnancies

Eighteen studies were identified that looked at the blood pressure response both during and following an acute bout of aerobic exercise in uncomplicated pregnancies.^51,73,75,77–79,82–84,87,89–96^ All studies reported an acute increase in SBP and DBP during aerobic exercise. One study comparing stationary cycling and treadmill walking found similar increases in SBP irrespective of the mode (bike: + 8 mmHg p = 0.06, treadmill: + 8 mmHg p = 0.02) and DBP (bike: + 5 mmHg p = 0.39, treadmill: + 6 mmHg p = 0.18).^51^ Fieril et al.^92^ also reported an increase in SBP and DBP following 15 and 30 min of aerobic exercise (p = 0.01 and p = 0.001 respectively). These two studies, along with De Olivieria et al.^91^ found a post exercise hypotensive response, in which BP dropped below baseline levels from 50 min to 14 hours post exercise.^51^ Two studies that observed BP responses to peak/max cycle tests found lower absolute BP responses in the first and second trimesters, increasing back to non-pregnant levels or above in the third trimester.^90,93^ One study found a positive correlation between resting SBP and DBP in the second trimester and BP response to submaximal aerobic exercise on the treadmill.^94^

#### At risk populations

Four studies measured acute BP response to aerobic exercise in at risk populations. The participants in two of these studies took part in an intervention of exercise during pregnancy however the authors reported acute BP responses to exercise rather than changes from baseline to post intervention.^41,76^ Mean SBP rose significantly after five minutes of exercise in one study from 149 mmHg (range 130 ± 170 mmHg) to 171 mmHg (range 150 ± 190 mmHg) in participants with pregnancy-induced hypertension.^80^ Diastolic BP also rose however was not significant in this study (102 mmHg, range 100 ± 110 mmHg to 106 mmHg, range 100 ± 115 mmHg).^80^ Another study^41^ found a significant difference in mean SBP (exercise: 1.81 ± 2.4 mmHg, control: 9.86 ± 2.87 mmHg p = 0.03) and DBP (exercise: -0.28 ± 1.57 mmHg. control: 7.78 ± 1.96 mmHg p = 0.002) changes after walking compared with pre-walking. A study comparing responses to aerobic and resistance exercise found no significant change in SBP and DBP from baseline following exercise, with the intervention group recording a significantly higher SBP during aerobic exercise than resistance (p = < 0.01).^76^ No significant differences were found in BP responses following exercise when groups with PE, GDM and Cholestasis were compared.^81^

### Acute resistance exercise

#### Uncomplicated pregnancies

Eight studies measured BP following an acute bout of resistance training during healthy pregnancy.^40,43,52,85,86,88,92,97^ The participants in two studies^40,43^ took part in resistance interventions described earlier under ‘Resistance Exercise Interventions – Healthy Populations’; however, the authors reported both acute and long-term responses to exercise.

Overall SBP and DBP increased significantly from baseline during exercise and returned to pre-exercise levels within 5 min following exercise, with four studies reporting no significant difference between pre and post BP.^40,43,52,92^ One study comparing pregnant and non-pregnant women found that the SBP, DBP and MAP responses during exercise were all lower (p = 0.03, 0.02, 0.01, respectively) within the pregnant group.^86^ In comparison, another study^85^ found no significant differences between SBP and DBP responses between pregnant and non-pregnant groups. One study compared BP responses to 40% 10RM resistance exercises with and without the use of the Valsalva manoeuvre and found a significantly increases MAP when the Valsalva manoeuvre was performed compared to free breathing due to significantly higher systolic (121 ± 15 mmHg vs 116 ± 12 mmHg, p = 0.001) and diastolic blood pressures (79 ± 8 mmHg vs 77 ± 8 mmHg, p = 0.02).^88^

#### At risk populations

Three studies found no difference between pre and post SBP or DBP following light^61,71^ and moderate to vigorous^76^ resistance exercise in at risk pregnant women.

#### Adherence

Adherence was reported in 21 of the 32 intervention studies, with varied results across the studies with both low (n = 7; 33%–62.5%)^20,42,56,57,60,68,98^ and high rates of adherence (n = 14; 75%–95%) reported.^34,36–38,44,53,54,57,62–66,70^ Yeo et al.^68^ found that adherence rates decreased over time, with their participants instructed to exercise five times per week and only completing on average 2.5–4.5 sessions per week. One study reported that 28 of the 69 participants in the intervention group completed less than 70% of the exercise sessions and were therefore excluded from the study.^55^

## Discussion

The aims of this review were to assess the effects of exercise interventions on blood pressure during pregnancy and to understand acute changes in blood pressure during a single bout of exercise in pregnant women. Significant differences in favour of the exercise group were found in SBP, DBP and MAP following exercise interventions in at-risk populations. This indicates that pregnant women at a higher risk for cardiovascular conditions may use aerobic or a combination of aerobic and resistance exercise to help prevent an increase in BP often associated with these conditions. For uncomplicated pregnancies, light to moderate intensity aerobic or resistance exercise had no effect on resting BP throughout pregnancy. Blood pressure showed greater increases with acute aerobic exercise than resistance exercise in uncomplicated and at-risk populations, returning to baseline levels post-exercise. A post-exercise hypotensive response in BP may occur following acute aerobic exercise, indicating that acute bouts of aerobic exercise may help lower BP in at risk populations with higher resting BP levels. Compared to usual care, aerobic, and/or resistance exercise performed throughout uncomplicated pregnancy had no influence on blood pressure; however, higher risk pregnancies may reduce their risk of elevated BP through regular exercise training during pregnancy.

This review found no differences in SBP or MAP in the uncomplicated pregnant population and only a small yet significant decrease in DBP following combined aerobic and resistance exercise intervention. Reassuringly, these participants remained normotensive throughout gestation. In response to vasoactive substances, growth factors and haemodynamic stimuli, the structural components of blood vessel walls are altered through the dynamic process of vascular remodelling during pregnancy.^4,14^ The structure and function of arteries are remodelled to accommodate an increased blood volume and cardiac output, and to ensure that the endothelial shear rates remain within healthy limits.^6,14^ A curvilinear reduction in blood pressure associated with vascular remodelling and vasodilation has been observed in uncomplicated pregnancies, with a nadir reached between the end of the first and beginning of the second trimester.^14,99^ The results from this meta-analysis support previous evidence which indicate that regular exercise during pregnancy does not influence these normal physiological changes that occur during gestation.^55^ Women with uncomplicated pregnancies can be confident that there are no adverse effects of exercise on haemodynamics during gestation. They should be encouraged to continue exercising throughout their pregnancy where possible.

The physiological changes present throughout gestation have been shown to differ between uncomplicated and pathological pregnancies.^2,4^ Where normal pregnancy is characterized by a low systemic vascular resistance and an increased cardiac output, the adaptations are often reversed in hypertensive pregnancies.^18,100,101^ Women with insulin resistance or GDM have an increased risk of developing GHTN and PE, and these conditions share several risk factors and pathophysiological features including maternal obesity, excessive gestational weight gain, vascular dysfunction, and inflammation.^22,54,76^ This review found exercising participants diagnosed with clinical conditions showed lower resting BP’s following intervention than the non-exercising controls, indicating that regular exercise may help prevent the onset of GHTN or PE in this population.^54,59^

The studies in this review that measured incidence of PE and GHTN identified significantly lower rates of these two conditions in exercising groups compared to non-exercising controls.^41,56,68^ Furthermore, no adverse events were reported by any of the interventions involving at-risk pregnancies, even those at high risk for GHTN and PE. This is supported by a systematic review which reported a 39% and 41% reduction in the odds of developing GHTN and a PE, respectively, when exercise was performed during pregnancy.^22^ Preeclampsia and GHTN have long been recognized as absolute and relative contraindications to exercise in international exercise and pregnancy guidelines.^102^ A review evaluating which clinical conditions may be contraindications to exercise determined that only severe PE should still be considered an absolute contraindication, with mild PE categorized as a relative contraindication, and gestational hypertension (in isolation) no longer considered a contraindication.^102^ The review highlighted that light to moderate prenatal exercise in women with mild pre-eclampsia caused no adverse changes in BP, uterine blood flow and FHR, and can provide a multitude of maternal and foetal benefits.^102^ It is crucial that pregnant women with these clinical conditions are provided with appropriate guidance based on the most recent evidence to improve maternal and foetal outcomes. More research is needed on the effects of exercise on BP regulation during pregnancy in those at a higher risk of developing gestational hypertensive conditions.^94^

Adherence appears to be a limitation in most studies involving overweight or obese pregnant women, with adherence rates between 33% and 75% reported in exercise interventions.^56,98^ Exercise adherence within at risk pregnant populations, particularly women who are overweight or have obesity, is considered a major challenge, therefore finding methods to reduce participant attrition rates is vital.^56^ It has been suggested that including higher intensity intervals into training may be one method of increasing energy expenditure while enhancing enjoyment levels and reducing the time spent exercising.^103^ Six of the studies included more vigorous intensity exercise,^34,56–58,63,73^ with adherence rates varying from 50%^56^ to 96%.^58^ Systematic evidence has found that vigorous intensity exercise appears safe for most uncomplicated pregnancies when completed into the third trimester,^30^ however further research is needed within the first and second trimester as well as within higher risk populations.

No significant differences in BP were found following resistance training alone, however only a limited number of studies reported the effects of resistance training during pregnancy. More research is needed on this modality of exercise throughout pregnancy to determine the long-term effects of resistance training on BP, specifically in at risk populations. Similar changes were seen with aerobic and combination exercise in both uncomplicated and at risk populations. It has previously been suggested that aerobic exercise should be supplemented with resistance exercise to aid in the prevention of hypertension in non-pregnant populations,^104,105^ however more recent evidence including a systematic review^106^ has identified that there is little to no difference in BP between aerobic exercise alone and a combination of aerobic and resistance in non-pregnant populations.^104,106^ The findings from this review suggest that within at risk populations aerobic and combination exercise should be prioritized to prevent an increase in BP and reduce the risk of developing gestational hypertensive conditions. Although resistance training may not significantly affect blood pressure changes throughout uncomplicated or at risk pregnancies, it is still recommended as standard exercise prescription due to the benefits to increase/maintain strength and decrease urinary incontinence.^107^

As expected, all of the acute studies found significant increases in SBP during exercise, with hypotensive BP responses found following aerobic exercise from 50 to 60 min^91^ to 13 to 14 h post exercise.^51^ Post exercise hypotension (PEH) is commonly seen following acute bouts of aerobic exercise in both normotensive and hypertensive non-pregnant people.^86,91^ Findings suggest that BP responses to acute aerobic exercise in pregnant women participating in regular aerobic exercise are significantly lower than non-exercising women. This indicates a training response to regular aerobic exercise with adaptations occurring within the cardiovascular system.^18^ Previous studies have suggested that some of the physiological mechanisms that reduce BP following chronic exercise may be present in the onset of PEH following acute exercise bouts. Indeed, a systemic adaptation of the arterial wall increasing arterial compliance occurs following an exercise session, thereby decreasing peripheral resistance.^18^ Characterized by a sustained decrease in blood pressure following a single bout of exercise, PEH has been shown to vary in magnitude and duration, indicating that exercise characteristics may have an influence on levels of PEH.^105,108^ It has been suggested that PEH responses are clinically important as they may help cause an adaptation which results in a lowering of BP.^43^ A reduction in SBP of as little as 2 mmHg in non-pregnant populations has been shown to reduce the risk of cardiovascular disease by 4-6%.^18^ The results of this review support previous research indicating that regular bouts of aerobic exercise may help pregnant women reduce their risk of developing gestational hypertensive conditions.

### Limitations

A limitation in the current review and meta-analysis was the heterogeneity of the research designs. A random effects meta-analysis was used to account for this. The *I*^2^ values were high for the uncomplicated and at risk groups when the exercise types were grouped (*I*^2^ = 94%–99%), and although they dropped slightly when subgroup analysis was performed for exercise type they remained high (*I*^2^ *=* 71%–98%) indicating that there may be heterogeneity in the outcomes that are not able to be explained by the studies in this systematic review. The leave-one-out analysis showed slight decreases in heterogeneity when certain studies were removed, however generally remained high (80%–99%). This can be expected as the session duration, intensity, frequency, exercise mode and length of intervention varied significantly across the studies, even within the subgroups presented (study variables can be viewed in Supplementary File 1. Tables S8 and S9). The mode, length (3–31 weeks), frequency (1–5 sessions/week), and duration (15–60 min), varied across interventions, making it hard to distinguish which of these factors may have contributed to changes in BP. A large decrease in heterogeneity was only seen when one study^53^ was removed. One notable difference in this study is that BP was measured through finger photoplethysmography with a Finometer (Finometer Pro; Finapres Medical Systems, Amsterdam, the Netherlands), rather than the more common method of brachial auscultation. Research has shown however, that the Finometer is a suitable measure of BP with no significant differences seen between auscultatory measures and Finometer measures when compared.^109^

The same issue was faced when comparing the acute studies, as the bouts ranged from 5- to 60-min bouts and were measured at different time points during pregnancy (12–38 weeks gestation). Most of the control groups were treated with routine prenatal care or continued with their usual physical activity levels, and as such may have participated in exercise throughout pregnancy of their own accord, potentially influencing results. Furthermore, there were low adherence rates and small sample sizes observed in many of the studies.

## Conclusion

The findings from this review indicate that moderate to vigorous aerobic exercise during pregnancies complicated with clinical conditions including GDM, overweight and obesity may either reduce, or attenuate an increase in blood pressure that commonly occurs with these conditions. These findings have important implications for pregnant women at risk of developing gestational hypertension and pre-eclampsia. Indeed, particular focus on providing exercise support to clinical pregnancies may have significant impact on future maternal and infant cardiovascular morbidity and mortality.

## Supplemental Material

sj-doc-4-whe-10.1177_17455057231183573 – Supplemental material for The effects of aerobic and resistance exercise on blood pressure in uncomplicated and at risk pregnancies: A systematic review and meta-analysis Click here for additional data file. Supplemental material, sj-doc-4-whe-10.1177_17455057231183573 for The effects of aerobic and resistance exercise on blood pressure in uncomplicated and at risk pregnancies: A systematic review and meta-analysis by Courtney Giles, Rich Johnston, Jade Kubler, Jemima Spathis and Kassia Beetham in Women’s Health

sj-docx-1-whe-10.1177_17455057231183573 – Supplemental material for The effects of aerobic and resistance exercise on blood pressure in uncomplicated and at risk pregnancies: A systematic review and meta-analysis Click here for additional data file. Supplemental material, sj-docx-1-whe-10.1177_17455057231183573 for The effects of aerobic and resistance exercise on blood pressure in uncomplicated and at risk pregnancies: A systematic review and meta-analysis by Courtney Giles, Rich Johnston, Jade Kubler, Jemima Spathis and Kassia Beetham in Women’s Health

sj-docx-2-whe-10.1177_17455057231183573 – Supplemental material for The effects of aerobic and resistance exercise on blood pressure in uncomplicated and at risk pregnancies: A systematic review and meta-analysis Click here for additional data file. Supplemental material, sj-docx-2-whe-10.1177_17455057231183573 for The effects of aerobic and resistance exercise on blood pressure in uncomplicated and at risk pregnancies: A systematic review and meta-analysis by Courtney Giles, Rich Johnston, Jade Kubler, Jemima Spathis and Kassia Beetham in Women’s Health

sj-docx-3-whe-10.1177_17455057231183573 – Supplemental material for The effects of aerobic and resistance exercise on blood pressure in uncomplicated and at risk pregnancies: A systematic review and meta-analysis Click here for additional data file. Supplemental material, sj-docx-3-whe-10.1177_17455057231183573 for The effects of aerobic and resistance exercise on blood pressure in uncomplicated and at risk pregnancies: A systematic review and meta-analysis by Courtney Giles, Rich Johnston, Jade Kubler, Jemima Spathis and Kassia Beetham in Women’s Health

## Declarations

**Ethics approval and consent to participate:** Not applicable.

**Consent for publication:** Not applicable.

**Availability of data and materials:** All supplementary files have been provided.
