# Supplementary material for: The effects of aerobic and resistance exercise on blood pressure in uncomplicated and at risk pregnancies: A systematic review and meta-analysis
Source: Womens Health (Lond). 2023 Jul 16;19:17455057231183573. doi: 10.1177/17455057231183573 (PMC10357069; doi:10.1177/17455057231183573)
Supplement: sj-docx-1-whe-10.1177_17455057231183573 – Supplemental material for The effects of aerobic and resistance exercise on blood pressure in uncomplicated and at risk pregnancies: A systematic review and meta-analysis [file sj-docx-1-whe-10.1177_17455057231183573.docx]

| **Supplementary File 1.** | | | | | |
| --- | --- | --- | --- | --- | --- |
| Search Strategy | | | | | |
| DATABASE | POPULATION |  | INTERVENTION |  | OUTCOME |
| CINAHL | Key Words:  pregnan* OR gestation* OR ''expect* mother'' OR ''expect* woman*'' OR ''expect* women*'' OR prenatal OR ''prenatal care'' OR antenatal OR perinatal OR peri-natal OR pre-natal OR peripartum OR peri-partum  Headings:  (MH "Pregnancy Trimesters") OR (MH "Pregnancy") OR (MH "Pregnancy Trimester, Third") OR (MH "Pregnancy Trimester, Second") OR (MH "Pregnancy Trimester, First") OR (MH "Expectant Mothers") OR (MH "Prenatal Care") OR (MH "Perinatal Care") | AND | Key Words:  exercise* OR exercising OR "physical activity" OR "physical activit*" OR "physical exert*" OR "physical fitness" OR "fitness train*" OR running OR sport* OR "physical train*" OR "exercis* therap*" OR “aerobic exercise*” OR conditioning OR "physical conditioning" OR “resistance train*” OR “strength train*” OR “resistance exercis*” OR “weight train*” OR “weight lift*” OR “power train” OR “powerlift*”  Headings:  (MH "Exercise") OR (MH "Therapeutic Exercise") OR (MH "Exercise Intensity") OR (MH "Plyometrics") OR (MH "Aerobic Exercises") OR (MH "Leisure Activities") OR (MH "Physical Activity") OR (MH "Physical Fitness") OR (MH "Sports") OR  (MH "Resistance Training") OR (MH "Weight lifting") OR (MH "Muscle Strengthening") | AND | Key Words:  MAP OR "mean arterial pressure" OR "blood pressure" OR BP OR "vascular *function" OR hypertensi* OR hypotensi* OR "high blood pressure" OR haemodynamic* OR hemodynamic* OR "cardiovascular function" OR vascular OR "central blood pressure" OR "aortic blood pressure"  Headings:  (MH "Blood Pressure") OR (MH "Hypertension") OR (MH "Hypotension") OR (MH "Arterial Pressure") OR (MH "Systolic Pressure") OR (MH "Hemodynamics") |
| MEDLINE | Key Words:  pregnan* OR gestation* OR ''expect* mother'' OR ''expect* woman*'' OR ''expect* women*'' OR prenatal OR ''prenatal care'' OR antenatal OR perinatal OR peri-natal OR pre-natal OR peripartum OR peri-partum  MeSH:  (MH "Pregnant Women") OR (MH "Pregnancy") OR (MH "Pregnancy Trimesters") OR (MH "Pregnancy Trimester, First") OR (MH "Pregnancy Trimester, Second") OR (MH "Pregnancy Trimester, Third") OR (MH "Prenatal Care") OR (MH "Perinatal Care") OR (MH "Peripartum Period") |  | Key Words:  exercise* OR exercising OR "physical activity" OR "physical activities" OR "physical exert*" OR "physical fitness" OR "fitness train*" OR running OR sport* OR "physical train*" OR "exercis* therap*" OR "aerobic exercise*” OR conditioning OR "physical conditioning" OR “resistance train*” OR “strength train*” OR “resistance exercis*” OR “weight train*” OR “weight lift*” OR “power train” OR “powerlift*”  MeSH:  (MH "Exercise") OR (MH "Plyometric Exercise") OR (MH "Exercise Therapy") OR (MH "Circuit-Based Exercise") OR (MH "High-Intensity Interval Training") OR (MH "Physical Conditioning, Human") OR (MH "Running") OR (MH "Leisure Activities") OR (MH "Physical Exertion") OR (MH "Physical Fitness") OR (MH "Cardiorespiratory Fitness") OR (MH "Sports") OR  (MH "Resistance Training") OR (MH "Weight Lifting") OR (MH "Muscle Strengthening") |  | Key Words:  MAP OR "mean arterial pressure" OR "blood pressure" OR BP OR "vascular *function" OR hypertensi* OR hypotensi* OR "high blood pressure" OR haemodynamic* OR hemodynamic* OR "cardiovascular function" OR vascular OR "central blood pressure" OR "aortic blood pressure"  Headings:  (MH "Blood Pressure") OR (MH "Arterial Pressure") OR (MH "Hypertension") OR (MH "Hypotension") OR (MH "Hemodynamics") |
| EMBASE | Key Words:  pregnan* OR gestation* OR ''expect* mother'' OR ''expect* woman*'' OR ''expect* women*'' OR prenatal OR ''prenatal care'' OR antenatal OR perinatal OR peri-natal OR pre-natal OR peripartum OR peri-partum  Emtree:  'pregnant woman'/exp OR 'pregnancy'/exp OR 'first trimester pregnancy'/exp OR 'second trimester pregnancy'/exp OR 'third trimester pregnancy'/exp OR 'expectant mother'/exp OR 'prenatal care'/exp |  | Key Words:  exercise* OR exercising OR 'physical activity' OR 'physical activities' OR 'physical exert*' OR 'physical fitness' OR 'fitness train*' OR running OR sport* OR 'physical train*' OR 'exercis* therap*' OR 'aerobic exercise*' OR conditioning OR 'physical conditioning' OR 'resistance train*' OR 'strength train*' OR 'resistance exercis*' OR 'weight train*' OR 'weight lift*' OR 'power train' OR 'powerlift*'  Emtree:  'exercise'/exp OR 'aerobic exercise'/exp OR 'circuit training'/exp OR 'exercise intensity'/exp OR 'high intensity interval training'/exp OR 'plyometrics'/exp OR 'dynamic exercise'/exp OR 'physical activity'/exp OR 'cycling'/exp OR 'running'/exp OR 'fitness'/exp OR 'kinesiotherapy'/exp OR 'movement therapy'/exp OR 'muscle training'/exp OR 'sport'/exp OR 'resistance training'/exp |  | Key Words:  MAP OR 'mean arterial pressure' OR 'blood pressure' OR BP OR 'vascular *function' OR hypertensi* OR hypotensi* OR 'high blood pressure' OR haemodynamic* OR hemodynamic* OR 'cardiovascular function' OR vascular OR 'central blood pressure' OR 'aortic blood pressure'  Emtree:  'blood pressure'/exp OR 'arterial pressure'/exp OR 'hemodynamics'/exp OR 'mean arterial pressure'/exp OR 'cardiovascular function'/exp |
| WEB OF SCIENCE | Key Words:  pregnan* OR gestation* OR ''expect* mother'' OR ''expect* woman*'' OR ''expect* women*'' OR prenatal OR ''prenatal care'' OR antenatal OR perinatal OR peri-natal OR pre-natal OR peripartum OR peri-partum |  | Key Words:  exercise* OR exercising OR "physical activity" OR "physical activities" OR "physical exert*" OR "physical fitness" OR "fitness train*" OR running OR sport* OR "physical train*" OR "exercis* therap*" OR ''aerobic exercise*'' OR conditioning OR "physical conditioning" OR “resistance train*” OR “strength train*” OR “resistance exercis*” OR “weight train*” OR “weight lift*” OR “power train” OR “powerlift*” |  | Key Words:  MAP OR "mean arterial pressure" OR "blood pressure" OR BP OR "vascular *function" OR hypertensi* OR hypotensi* OR "high blood pressure" OR haemodynamic* OR hemodynamic* OR "cardiovascular function" OR vascular OR "central blood pressure" OR "aortic blood pressure" |
| PUBMED | Key Words:  pregnan* OR gestation* OR ''expect* mother'' OR ''expect* woman*'' OR ''expect* women*'' OR prenatal OR ''prenatal care'' OR antenatal OR perinatal OR peri-natal OR pre-natal OR peripartum OR peri-partum  MeSH:  "Pregnant Women"[Mesh] OR "Pregnancy"[Mesh] OR "Pregnancy Trimesters"[Mesh] OR "Pregnancy Trimester, Third"[Mesh] OR "Pregnancy Trimester, Second"[Mesh] OR "Pregnancy Trimester, First"[Mesh] OR "Prenatal Care"[Mesh] OR "Peripartum Period"[Mesh] OR "Perinatal Care"[Mesh] |  | Key Words:  exercise* OR exercising OR "physical activity" OR "physical activities" OR "physical exert*" OR "physical fitness" OR "fitness train*" OR running OR sport* OR "physical train*" OR "exercis* therap*" OR "aerobic exercise*" OR conditioning OR "physical conditioning" OR “resistance train*” OR “strength train*” OR “resistance exercis*” OR “weight train*” OR “weight lift*” OR “power train” OR “powerlift*”  MeSH:  "Exercise"[Mesh] OR "Exercise Therapy"[Mesh] OR “High-Intensity Interval Training"[Mesh] OR "Physical Exertion"[Mesh] OR "Physical Fitness"[Mesh] OR "Running"[Mesh] OR "Sports"[Mesh] OR "Physical Conditioning, Human"[Mesh] OR "Plyometric Exercise"[Mesh] OR "Circuit-Based Exercise"[Mesh] OR "Leisure Activities"[Mesh] OR "Resistance Training"[Mesh] OR "Exercise Movement Techniques"[Mesh] |  | Key Words:  MAP OR "mean arterial pressure" OR "blood pressure" OR BP OR "vascular *function" OR hypertensi* OR hypotensi* OR "high blood pressure" OR haemodynamic* OR hemodynamic* OR "cardiovascular function" OR vascular OR "central blood pressure" OR "aortic blood pressure"  MeSH:  "Blood Pressure"[Mesh] OR "Arterial Pressure"[Mesh] OR "Hypotension"[Mesh] OR “Hypertension"[Mesh] OR "Hemodynamics"[Mesh] |
| COCHRANE | Key Words:  pregnan* OR gestation* OR ''expect* mother'' OR ''expect* woman*'' OR ''expect* women*'' OR prenatal OR ''prenatal care'' OR antenatal OR perinatal OR peri-natal OR pre-natal OR peripartum OR peri-partum  MeSH:  (Pregnant Women) OR (Pregnancy) OR (Pregnancy Trimesters) OR (Pregnancy Trimester, First) OR (Pregnancy Trimester, Second) OR (Pregnancy Trimester, Third) OR (Prenatal Care) |  | Key Words:  exercise* OR exercising OR "physical activity" OR "physical activities" OR "physical exert*" OR "physical fitness" OR "fitness train*" OR running OR sport* OR "physical train*" OR "exercis* therap*" OR "aerobic exercise*" OR conditioning OR "physical conditioning" OR “resistance train*” OR “strength train*” OR “resistance exercis*” OR “weight train*” OR “weight lift*” OR “power train” OR “powerlift*”  MeSH:  (Exercise) OR (Exercise Therapy) OR (Plyometric Exercise) OR (Circuit-Based Exercise) OR (High-Intensity Interval Training) OR (Physical Conditioning, Human) OR (Running) OR (Leisure Activities) OR (Physical Exertion) OR (Physical Fitness) OR (Cardiorespiratory Fitness) OR (Sports) OR (Resistance Training) OR (Weight Lifting) |  | Key Words:  MAP OR "mean arterial pressure" OR "blood pressure" OR BP OR "vascular *function" OR hypertensi* OR hypotensi* OR "high blood pressure" OR haemodynamic* OR hemodynamic* OR "cardiovascular function" OR vascular OR "central blood pressure" OR "aortic blood pressure"  MeSH:  (Blood Pressure) OR (Arterial Pressure) OR (Hypotension) OR (Hypertension) OR (Hemodynamics) |
