# Supplementary material for: The effects of aerobic and resistance exercise on blood pressure in uncomplicated and at risk pregnancies: A systematic review and meta-analysis
Source: Womens Health (Lond). 2023 Jul 16;19:17455057231183573. doi: 10.1177/17455057231183573 (PMC10357069; doi:10.1177/17455057231183573)
Supplement: sj-docx-2-whe-10.1177_17455057231183573 – Supplemental material for The effects of aerobic and resistance exercise on blood pressure in uncomplicated and at risk pregnancies: A systematic review and meta-analysis [file sj-docx-2-whe-10.1177_17455057231183573.docx]

**Supplementary File 2.**

Table S1. Cochrane Risk of Bias for Randomised Controlled Trials

|  | Selection Bias (random sequence generation) | Selection Bias (allocation concealment) | Reporting Bias | Other Bias | Performance Bias | Detection Bias | Attrition Bias | Overall |
| --- | --- | --- | --- | --- | --- | --- | --- | --- |
| Amorim, 2018, Brazil | L | L | L | L | L | U | U | L |
| Babbar, 2016, USA | L | L | L | L | L | L | L | L |
| Bahadoran, 2015 Iran | U | H | U | U | U | U | U | U |
| Barakat, 2011, Spain | L | U | U | U | L | L | L | L |
| Barakat, 2012, Spain | L | L | U | U | L | L | L | L |
| Barakat, 2014, Spain | L | L | U | U | U | L | L | L |
| Boparai, 2021, Canada | L | L | L | L | L | L | U | L |
| Brislane, 2021, UK | H | H | L | U | L | L | U | U |
| Brun, 2011, Canada | U | U | U | U | L | L | U | U |
| Carpenter, 2015, UK | H | U | U | U | U | L | U | U |
| Carpenter, 2017, UK | H | U | U | U | L | U | U | U |
| Daniel, 2015, Nigeria | L | U | U | L | L | L | U | L |
| de Oliveria, 2012, Brazil | L | L | L | L | L | L | L | L |
| Erkkola, 1976, Finland | U | U | U | L | L | L | U | U |
| Fernández-Buhigas, 2020, Spain | L | L | U | U | L | L | U | L |
| Garnaes, 2016, Norway | L | L | L | L | L | L | L | L |
| Guelfi, 2016, Australia | L | L | L | U | L | L | U | L |
| Haakstad, 2016, Norway | L | L | L | L | L | L | L | L |
| Halse, 2015, Australia | L | L | L | L | U | L | U | L |
| Huifen, 2022, China | L | L | L | L | L | L | L | L |
| Kasawara, 2013, Brazil | L | L | L | L | L | L | L | L |
| Khoram, 2019, Iran | L | L | L | L | U | L | L | L |
| Kim, 2018, Korea | L | L | L | L | L | L | L | L |
| Nascimento, 2011, Brazil | L | L | L | L | L | L | L | L |
| Perales, 2016, Spain | L | L | L | L | L | L | L | L |
| Petrov Fieril, 2015, Sweden | L | L | L | L | L | L | L | L |
| Pijpers, 1984, Netherlands | U | U | U | U | U | L | U | U |
| Ramirez-Velez, 2011, Columbia | L | L | L | L | L | L | L | L |
| Rodríguez-Díaz, 2017, Spain | L | L | L | U | L | L | L | L |
| Seneviratne, 2015, New Zealand | L | L | L | L | L | U | L | L |
| Silva-Jose, 2021, Spain | L | L | L | L | L | L | L | L |
| Sklempe Kokic, 2018, Croatia | U | U | U | U | L | L | U | U |
| Stutzman, 2010, Canada | H | H | L | L | L | L | U | L |
| Vladimirov, 2015, Poland | L | L | U | U | U | L | L | L |
| Webb, 1994, Canada | H | H | H | H | U | U | U | H |
| Yeo, 2000, USA | U | U | L | U | L | U | L | U |
| Yeo, 2008, USA | U | U | L | L | L | U | L | L |

Table S2. Newcastle-Ottawa Scale for Cohort Studies

|  | Representativeness | Selection | Ascertainment of exposure | Demonstration that outcome of interest was not present at start | Study controls for relevant primary confounder | Study controls for other secondary confounders | Assessment of outcome | Was follow up long enough for outcomes to occur | Adequacy of follow up of cohorts | Total |
| --- | --- | --- | --- | --- | --- | --- | --- | --- | --- | --- |
| Pivnarik, 1993, USA | 1 | 1 | 0 | 1 | 1 | 0 | 0 | 1 | 1 | 6 |
| Rafla, 1999, UK | 0 | 0 | 1 | 1 | 0 | 0 | 1 | 1 | 1 | 5 |
| Rafla, 2000, UK | 0 | 1 | 1 | 1 | 0 | 0 | 1 | 1 | 1 | 6 |
| Rauramo, 1988, Finland | 0 | 1 | 1 | 1 | 0 | 0 | 1 | 1 | 1 | 6 |
| Rauramo, 1988, Finland | 0 | 0 | 0 | 1 | 1 | 0 | 0 | 1 | 1 | 4 |
| Sady, 1990, USA | 0 | 0 | 0 | 1 | 1 | 0 | 0 | 1 | 1 | 4 |
| Santos, 2016, Brazil | 1 | 1 | 0 | 1 | 1 | 0 | 0 | 1 | 1 | 6 |

Table S3. Newcastle-Ottawa Scale for Case-Control Studies

|  | Adequate case definition | Representativeness of the Cases | Selection of Controls | Definition of Controls | Comparability of Cases and Controls on the basis of the Design or Analysis | Ascertainment of Exposure | Non-Response Rate | Total |
| --- | --- | --- | --- | --- | --- | --- | --- | --- |
| Avery, 1999, Canada | 1 | 1 | 1 | 0 | 1 | 1 | 1 | 6 |
| Bgeginski, 2015, Brazil | 1 | 1 | 1 | 0 | 1 | 1 | 1 | 6 |
| Meah, 2021, Canada | 1 | 1 | 1 | 1 | 1 | 0 | 1 | 6 |
| Meah, 2021, Canada | 1 | 1 | 1 | 1 | 1 | 0 | 1 | 6 |
| O'Neill, 1993, Australia | 1 | 0 | 1 | 0 | 1 | 1 | 1 | 5 |
| Purdy, 2019, Canada | 1 | 0 | 1 | 0 | 1 | 1 | 1 | 5 |

Table S4. ROB-2 Tool assessment of risk of bias in Crossover Trials

|  | *Randomisation process* | *Period/Carryover effects* | *Effect of assignment to intervention* | *Adhering to intervention* | *Missing outcome data* | *Measurement of the outcome* | *Selection of the reported result* |  |
| --- | --- | --- | --- | --- | --- | --- | --- | --- |
|  |  |  |  |  |  |  |  |  |
|  |  |  |  |  |  |  |  |  |
|  |  |  |  |  |  |  |  | *Total* |
| *Petrov Fieril, 2016, Sweden* | H | L | L | L | L | L | L | L |
| *de Oliveria, 2014, Brazil* | SC | L | L | L | L | L | L | L |

Table S5. Assessment of risk of bias in Single-arm clinical trials

|  | Q1 | Q2 | Q3 | Q4 | Q5 | Total |
| --- | --- | --- | --- | --- | --- | --- |
| Bisson, 2014, Canada | L | L | L | L | U | L |
| Ferriera, 2014, Brazil | L | L | L | L | L | L |
| Jeffreys, 2006, USA | L | L | L | L | U | L |
| Morrow et al. (1989) | H | L | L | L | U | L |
| O'Connor et al. (2011) | L | L | L | L | L | L |
| O'Neill et al. (2006) | L | U | L | L | L | L |
| van Doorn et al. (1992) | U | U | L | U | U | U |
