# Supplementary material for: The effects of aerobic and resistance exercise on blood pressure in uncomplicated and at risk pregnancies: A systematic review and meta-analysis
Source: Womens Health (Lond). 2023 Jul 16;19:17455057231183573. doi: 10.1177/17455057231183573 (PMC10357069; doi:10.1177/17455057231183573)
Supplement: sj-docx-3-whe-10.1177_17455057231183573 – Supplemental material for The effects of aerobic and resistance exercise on blood pressure in uncomplicated and at risk pregnancies: A systematic review and meta-analysis [file sj-docx-3-whe-10.1177_17455057231183573.docx]

Supplementary File 3

Table S6. Characteristics of Intervention Studies

| **Author, Year, Country** | **Population**  **Healthy or Clinical**  **(Clinical conditions)** | **Subjects (n)** | | | **GA (Weeks)** | **Baseline Measures** | | | | | | | **Control** | | | | | | **Outcomes** | |
| --- | --- | --- | --- | --- | --- | --- | --- | --- | --- | --- | --- | --- | --- | --- | --- | --- | --- | --- | --- | --- |
|  |  | **Total    Int   Con** | | |  | **Age**  **(years)** | **BMI (kg/m^2^) or Weight (kg)** | | **SBP**  **(mmHg)** | **DBP (mmHg)** | **MAP**  **(mmHg)** | **Age**  **(years)** | | **BMI (kg/m^2^) or Weight (kg)** | **SBP**  **(mmHg)** | **DBP (mmHg)** | **MAP**  **(mmHg)** |  | |  |
| RCTs | | | | | | | | | | | | | | | | | | | | |
| Barakat, 2011, Spain | Uncomplicated | 67 | 34 | 33 | 6-9 | 31 ± 3 | 23.9 ± 3 | NR | | NR | NR | 30 ± 3 | | 24.8 ± 4 | NR | NR | NR | No significant differences | |  |
| Barakat, 2012, Spain | Uncomplicated | 290 | 152 | 138 | 6-9 | 31.4 ± 3.2 | 24 ± 4.3 | NR | | NR | NR | 31.7 ± 4.5 | | 23.6 ± 4 | NR | NR | NR | No Significant differences | |  |
| Barakat, 2014, Spain | Uncomplicated | 200 | 107 | 93 | 9-13 | 31.57 ± 3.87 | 23.78 ± 4.4 | NR | | NR | NR | 31.51 ± 3.92 | | 24.09 ± 4.32 | NR | NR | NR | No significant differences | |  |
| Boparai, 2021, Canada | Uncomplicated | 27 | 16 | 11 | 16-20 | 32.6 ± 0.9 | 28.9 ± 6.6 | 115 ± 12 | | 66 ± 9 | 85 ± 9 | 31 ± 0.7 | | 25.5 ± 3 | 107 ± 11 | 64 ± 9 | 81 ± 8 | Int: ↓ SBP  Con: ↑ SBP | |  |
| Brislane, 2021, UK | Uncomplicated | 18 | 7 | 11 | <12 | 33 ± 4 | 23 ± 3 | 99 ± 6 | | 60 ± 8 | Calc.  73 ± 8.54 | 33 ± 3 | | 24 ± 3 | 103 ± 12 | 60 ± 6 | Calc. 74.33 ± 8.49 | No significant changes | |  |
| Carpenter, 2015, UK | Uncomplicated | 50 | 34 | 16 | 20 | 26.4 ± 1.3 | NR | 105.8 ± 1.9 | | 72.3 ± 2.8 | Calc. 80.6 ± 7.34 | 24.6 ± 0.7 | | NR | 109 ± 1.7 | 70.6 ± 1.2 | Calc. 83.4 ± 7.75 | Int: ↑ SBP, DBP | |  |
| Carpenter, 2017, UK | Uncomplicated | 51 | 16 | 35 | 20 | 19-24 n=3 25-29 n=4  30-34 n=6  35-39 n=2  40+ n=1 | 18.5-24.9 n=8; 25-29.9 n=2; >30 n=6 | 105.8 ± 1.9 | | NR | NR | 19-24 n=2 25-29 n=12  30-34 n=12  35-39 n=5 | | 18.5-24.9 n=23; 25-29.9 n=6; >30 n=6 | 109 ± 1.7 | NR | NR | No significant differences | |  |
| Daniel, 2015, Nigeria | At risk (GDM) | 30 | 15 | 15 | 24-28 | 32 ± 3.43 | 82.77 ± 14.62 kg | 108.67 ± 8.84 | | 77.4 ± 11.54 | Calc. 87.82 ± 10.72 | 32.93 ± 4.61 | | 85.23 ± 8.14 kg | 110.67 ± 7.68 | 76.48 ± 10.63 | Calc. 87.88 ± 9.75 | Int: ↓ SBP, DBP | |  |
| de Oliveria, 2012, Brazil | Uncomplicated | 187 | 125 | 62 | 13-20 | 24 ± 4 | 25 ± 5.5 | NR | | NR | NR | 23.5 ± 3.5 | | 24 ± 5.4 | NR | NR | NR | No significant differences | |  |
| Erkkola, 1976, Finland | Uncomplicated | 62 | 30 | 32 | 10-14 | 23.6 ± 1.7 | 57.5 ± 6.4 | 126.8 ± 14.1 | | 76.2 ± 9.1 | Calc. 93.07 ± 11.02 | 23.2 ± 1.7 | | 58.4 ± 6 | 124.4 ± 12.4 | 74.4 ± 7.7 | Calc. 91.07 ± 9.53 | No significant differences | |  |
| Fernández-Buhigas, 2020, Spain | Uncomplicated | 92 | 41 | 51 | <16 | 33.17 ± 3.19 | 22.81 ± 3.54 | 120.54 ± 10.56 | | 72.65 ± 8.7 | Calc. 88.61 ± 9.34 | 32.63 ± 4.66 | | 23.8 ± 5.09 | 119.51 ± 11.26 | 73.05 ± 7.2 | Calc. 88.54 ± 8.76 | No significant differences | |  |
| Garnæs, 2016, Norway | At risk (Obese BMI >28kg/m^2^) | 74 | 38 | 36 | 12-18 | 31.3 ± 3.8 | 33.9 ± 3.8 | 126.3 ± 20.9 | | 75.0 ± 10.0 | Calc. 92.1 ± 9.6 | 31.4 ± 4.7 | | 35.1 ± 4.6 | 127.9 ± 12.9 | 78.0 ± 8.4 | Calc. 94.63 ± 9.73 | Con: ↑ SBP | |  |
| Guelfi, 2016, Australia | At risk (History of GDM) | 157 | 81 | 76 | <14 | 33.6 ± 4.1 | 26.3 ± 5.1 | 106 ± 11 | | 63 ± 8 | Calc. 77.33 ± 8.79 | 33.8 ± 3.9 | | 25.7 ± 5.4 | 106 ± 13 | 64 ± 9 | Calc. 78 ± 10.5 | No significant differences | |  |
| Haakstad, 2016, Norway | Uncomplicated | 61 | 35 | 26 | 12-24 | 31.5 ± 3.1 | 22.9 ± 3.2 | 115 ± 12 | | 66 ± 7 | Calc. 82.33 ± 8.98 | 29.4 ± 3.8 | | 23.0 ± 3.1 | 115 ± 10 | 67 ± 9 | Calc. 83 ± 9.35 | Con: ↑ SBP, DBP | |  |
| Halse, 2015, Australia | At risk  (GDM) | 40 | 20 | 20 | 26-30 | 34 ± 5 | 25.2 ± 6.7 | 107 ± 17 | | 66 ± 11 | Calc. 79.67 ± 8.93 | 32 ± 3 | | 26.4 ± 7.1 | 110 ± 7 | 70 ± 9 | Calc. 83.33 ± 8.39 | No significant differences | |  |
| Huifen, 2022, China | At risk (GDM) | 89 | 43 | 46 | 28.02 ± 2.01 | 31.84 ± 5.19 | 23.03 ± 5.22 | 121.37 ± 15.83 | | 75.63 ± 8.96 | Calc. 90.90 ± 11.71 | 31.35 ± 4.72 | | 21.98 ± 2.96 | 119.8 ± 17.47 | 75.65 ± 10.86 | Calc. 90.37 ± 13.4 | Int: ↓ SBP, DBP | |  |
| Kasawara, 2013, Brazil | At risk (chronic HTN/previous PE) | 109 | 56 | 53 | 12-20 | <19 n=1  20-29 n=21  30-39 n=27  ≥ 40 n=9 | 18.5–24.9 n=4  25–29.9 n=13 30–39.9 n=26 ≥40 n=15 | 116 ± 16.9 | | 74.6 ± 15 | Calc. 88.4 ± 14.42 | <19 n=1  20-29 n=20  30-39 n=31  ≥ 40 n=6 | | 18.5–24.9 n=6  25–29.9 n=11 30–39.9 n=31 ≥40 n=10 | 120.6 ± 13.9 | 77.1 ± 13.2 | Calc. 91.6 ± 13.44 | No significant changes | |  |
| Khoram, 2019, Iran | At risk (chronic HTN, previous PE, Hx of GHT, FHx of HTN | 72 | 36 | 36 | 14 | 31.91 ± 4.62 | 27.36 ± 3.64 | NR | | NR | NR | 31± 5.29 | | 34.97 ± 4.77 | NR | NR | NR | Int: ↓ SBP. DBP | |  |
| Nascimento, 2011, Brazil | At risk (Overweight/ Obese BMI >26kg/m^2^) | 80 | 39 | 41 | 14-24 | 29.7 ± 6.8 | 34.8 ± 6.6 | NR | | NR | NR | 30.9 ± 5.9 | | 36.4 ± 6.9 | NR | NR | NR | No significant changes | |  |
| Perales, 2016, Spain | Uncomplicated | 241 | 121 | 120 | 9-11 | 31 ± 4 | 23.8 ± 4.2 | 115.5 ± 13.0 | | 65.0 ± 10.3 | Calc. 81.83 ± 11.27 | 31 ± 4 | | 25.1 ± 4.7 | 115.6 ± 12.4 | 66.9 ± 11.4 | Calc. 83.1 ± 11.96 | No significant changes | |  |
| Petrov Fieri, 2015, Sweden | Uncomplicated | 72 | 38 | 34 | 13 | 30.8 ± 3.6 | 22.6 ± 2.5 | 109 ± 18.8 | | 66.2 ± 8.3 | Calc. 80.47 ± 12.8 | 30.6 ± 3.4 | | 23.0 ± 2.6) | 111 ± 10.5 | 63.7 ± 7.7 | Calc. 79.47 ± 8.73 | No significant changes | |  |
| Ramirez-Velez, 2011, Columbia | Uncomplicated | 50 | 24 | 36 | 16-20 | 19.5 ± 2.3 | NR | 110.2 ± 10.6 | | 66.3 ± 10.4 | 81.0 ± 9.6 | 19.5 ± 2.3 | | NR | 109.8 ± 11.4 | 64.5 ± 6.8 | 79.6 ± 7.9 | No significant changes | |  |
| Rodríguez-Díaz, 2017, Spain | Uncomplicated | 105 | 50 | 55 | 26-28 | 32.87 ± 4.46 | 28.79 ± 4.27 | 108.72 ± 10.37 | | 65.63 ± 7.33 | Calc. 79.99 ± 8.47 | 31.52 ± 4.95 | | 26.78 ± 5.04 | 107.18 ± 10.08 | 65.16 ± 6.34 | Calc. 79.17 ± 7.79 | Int: ↓ SBP, DBP | |  |
| Seneviratne, 2015, New Zealand | At risk (Overweight/ Obese BMI >25kg/m^2^) | 75 | 38 | 37 | 20 | 31.6 ± 4.6 | 32.1 ± 4.4 | 113.2 ± 12.2 | | 67.8 ± 8.3 | Calc. 82.93 ± 9.11 | 31.1 ± 5.2 | | 34.1 ± 5.9 | 118.5 ± 9.8 | 70.0 ± 8.7 | Calc. 86.17 ± 9.28 | No significant changes | |  |
| Stutzman, 2010, Canada | Uncomplicated & at risk (Overweight/ Obese BMI >25kg/m^2^) | 22 | 11 | 11 | 18-22 | 30.4 ± 4.2 | 22.2 ± 1.7 | 111 ± 12 | | 76 ± 11 | Calc. 87.67 ± 11.34 | 20.9 ± 2.3 | | 25.8 ± 3 | 109 ± 7 | 74 ± 4 | Calc. 85.67 ± 5.2 | No significant changes | |  |
|  |  |  |  |  |  | 30.6 ± 5.5 | 28.8 ± 6.9 | 114 ± 14 | | 75 ± 10 | Calc. 88 ± 11.49 | 30.6 ± 4 | | 26.2 ± 5.6 | 107 ± 8 | 72 ± 4 | Calc. 83.67 ± 5.66 | Con: ↑ SBP, DBP | |  |
| Vladimirov, Poland, 2015 | At risk (Anaemia) | 88 | 50 | 38 | 20-27 | 25 ± 4.3 | NR | 135.91 ± 3.9 | | 83.09 ± 3.49 | Calc. 100.7 ± 10.03 | 25 ± 4.3 | | NR | 136.32 ± 2.8 | 82.8 ± 4.93 | Calc. 100.64 ± 4.34 | Int: ↓ SBP, DBP | |  |
| Yeo, 2000, USA | At risk (mild hypertension, Hx of GHT, FHx of HTN) | 16 | 8 | 8 | 18 | 30 ± 5.4 | NR | 109 | | 69 | Calc. 82.3 | 30 ± 5.4 | | NR | 109 | 69 | Calc. 82.3 | No significant changes | |  |
| **Clinical Trials** | | | | | | | | | | | | | | | | | | | | |
| Yeo, 2008, USA | At risk (Previous PE) | 79 | 41 | 38 | 18 | NR | NR | 106 | | 62 | Calc. 76.67 ± 8.61 | NR | | NR | 106 | 62 | Calc. 76.67 ± 8.26 | Con: ↑ SBP | |  |
| Ferriera, 2014, Brazil | Uncomplicated | 27 | 27 | 0 | 18 | 23.3 | 23.4 | 108.0 ± 13.5 | | 66.8 ± 10.1 | NR | N/A | | N/A | N/A | N/A | N/A | No significant changes | |  |
| O'Connor, 2011, USA | Uncomplicated | 32 | 32 | 0 | 21-25 | 29 ± 4 | 76 ± 2 kg | 166 ± 6 | | 113.5 ± 8.4 | 71.9 ± 6.8 | N/A | | N/A | N/A | N/A | N/A | No significant changes | |  |
| Silva-Jose, 2021, Spain | Uncomplicated | 72 | 31 | 41 | 8-10 | 32.29 ± 6.36 | 22.61 ± 3.22 | 110.55 ± 12.13 | | 71 ± 7.41 | NR | 33.93 ± 4.49 | | 23.06 ±7.8 | 110.76 ± 13.3 | 72.95 ± 8.07 | NR | No significant differences | |  |
| **Quasi-experimental Controlled Trial** | | | | | | | | | | | | | | | | | | | | |
| Bahadoran, 2015 Iran | Uncomplicated | 88 | 29 | 59 | 18-22 | 26.1 ± 3.27 | 23.9 ± 3.37 | 107.9 ± 10.39 | | 69.3 ± 8.31 | Calc. 81.5 ± 8.15 | 27.0 ± 3.57 | | 22.9 ± 2.97 | 106.9 ± 12.30 | 67.0 ± 6.77 | Calc. 80.3 ± 9 | No significant changes | |  |

*RCT* Randomised Control Trial, *GA* gestational age, *PA* physical activity, *NR* not reported, *GDM* gestational diabetes mellitus, *HTN* hypertension, *BMI* Body Mass Index, *PE* preeclampsia, *Hx* history *FHx* family history, *GHTN* gestational hypertension, *Con* control, *Int* intervention, *Ex* exercising, *GA* gestational age, *Calc* calculated

Table S7. Characteristics of Acute Studies

| **Author, Year, Country** | **Population**  **Healthy or Clinical**  **(clinical conditions)** | **Subjects (n)** | | | **Recruitment (GA weeks)** | **BMI, SBP, DBP, Age** | | | | | | | | **Control, BMI, SBP, DBP, Age** | | | | |
| --- | --- | --- | --- | --- | --- | --- | --- | --- | --- | --- | --- | --- | --- | --- | --- | --- | --- | --- |
|  |  | **Total    Int    Con** | | |  | **Age**  **(years)** | | | **BMI (kg/m^2^) or Weight (kg)** | | **SBP**  **(mmHg)** | | **DBP (mmHg)** | **Age**  **(years)** | **BMI (kg/m^2^) or Weight (kg)** | **SBP**  **(mmHg)** | | **DBP (mmHg)** |
| **RCTs** | | | | | | | | | | | | | | | | | | |
| Babbar, 2016, USA  Table 7. Characteristics of acute studies included in the systematic review | Uncomplicated | 46 | 23 | 23 | 28-36 | 25.5 ± 4.4 | | | 26.5 ± 6.1 | | 108 ± 12 | | 70.7 ± 6.9 | 25.4 ± 4.6 | 25.1 ± 6.7 | 106.4 ± 6.3 | | 69.3 ± 4.7 |
| Brun, 2011, Canada  Table 7. Characteristics of acute studies included in the systematic review | At risk (GDM) | 11 | 6 | 5 | 31 ± 3.5 | 30 ± 3.8 | | | NR | | 116 ± 4 | | 74 ± 4 | 30 ± 3.8 | NR | 115 ± 3 | | 71 ± 2.5 |
| Kim, 2018, Korea | At risk | 45 | 23 | 22 | >24 | 32.22 ± 2.58 | | | NR | | 107.39 ± 11.37 | | 67.83 ± 9.02 | 31.50 ± 4.48 | NR | 107.73 ± 10.20 | | 66.36 ± 10.93 |
| Pijpers, 1984, Netherlands | Uncomplicated | 23 | 11 | 12 | 34-38 | 26.1 | | | 66.1 kg | | 114.0 ± 6.8 | | 65.7 ± 4.0 | 26.1 | 66.1 kg | 119.2 ± 8.9 | | 72.4 ± 6.9 |
| Sklempe Kokic, 2018, Croatia | At risk (GDM) | 18 | 9 | 9 | 25.6 ± 5.2 | 32.8 ± 3.8 | | | 24.4 ± 4.9 | | 112.1 ± 7.1 | | 71.2 ± 6 | NA | NA | NA | | NA |
| Webb, 1994, Canada | Uncomplicated | 38 | 22 | 16 | 14-18 | 30.2 ± 0.9 | | | 68.7 ± 2.5 kg | | 145 ± 3 | | 74 ± 1 | 29.1 ± 0.9 | 63.3 ± 1.2 kg | | 143 ± 2 | 75 ± 1 |
| **Clinical Trials** | | | | | | | | | | | | | | | | | | |
| Amorim 2018, Brazil | Uncomplicated | 120 | 120 | N/A | 34-38 | Bike | | | | | | | | NA | NA | | NA | NA |
|  |  |  |  |  |  | 25 ± 6.4 | | | 32.2 ± 10.6 | | 110 | | 70 |  |  |  |  |  |
|  |  |  |  |  |  | Treadmill | | | | | | | |  |  |  |  |  |
|  |  |  |  |  |  | 25.2 ± 6.4 | | | 33.5 ± 8.7 | | 112 | | 70 |  |  |  |  |  |
| Avery, 1999, Canada  Table 7. Characteristics of acute studies included in the systematic review | Uncomplicated | 24 | 12 | NPC 12 | 30-32 | 29 ± 1 | | | 70 ± 3 kg | | NR | | NR | 29 ± 2 | 62 ± 2 kg | |  |  |
| Bgeginski, 2015, Brazil | Uncomplicated | 20 | 10 | NPC 10 | 22-24 | 25.3 ± 4.44 | | | 23.53 ± 2.48 | | NR | | NR | 25.2 ± 3.73 | 23.57 ± 2.59 | | NR | NR |
| Jeffreys, 2006, USA | Uncomplicated | 14 | 14 | NA | 31 ± 2 | 34 ± 3 | | | 24.5 ± 2.8 | | 110 ± 12 | | 67 ± 10 | NA | NA | | NA | NA |
| Meah, 2021, Canada | Uncomplicated | 30 | 15 | NPC 15 | 22.9 ± 5.9 | 33 ± 3 | | | 75 ± 27 | | 109 ± 15 | | 71 ± 10 | NPC  32 ± 8 | NPC  64 ± 8 | | NPC  106 ± 9 | NPC  71 ± 7 |
| Meah, 2021, Canada | Uncomplicated | 45 | 14 | NPC 18  PPC 13 | 22-26 | 32±3 | | | 26 ± 4 | | 109 ± 8 | | 63 ± 5 | NPC  28 + 4  PPC 33 ± 2 | NPC 23 ± 4 PPC 23 ± 4 | | NPC 113 ± 7  PPC 105 ± 6 | NPC 68 ± 6  PPC 61 ± 4 |
| Morrow, 1989, Canada  Table 7. Characteristics of acute studies included in the systematic review | Uncomplicated | 15 | 15 | N/A | 36-41 | NR | | | NR | | NR | | NR | NA | NA | | NA | NA |
| O'Neill, 2006, Aus | Uncomplicated | 50 | 50 | N/A | 34-40 | Semi recumbent | | | | | | | | NA | NA | | NA | NA |
|  |  |  |  |  |  | 30 ± 5 | | | 71 ± 9 | | 110 ± 9 | | 68 ± 8 |  |  |  |  |  |
|  |  |  |  |  |  | Upright | | | | | | | |  |  |  |  |  |
|  |  |  |  |  |  | 30 ± 4 | | | 69 ± 5 | | 107 ± 8 | | 70 ± 6 |  |  |  |  |  |
| O'Neill, 1993, Aus  Table 7. Characteristics of acute studies included in the systematic review | Uncomplicated | 39 | 39 | N/A | 23-28 | Trained | | | | | | | | NA | NA | | NA | NA |
|  |  |  |  |  |  | 30.1 ± 2.9 | | | 68.1 ± 6 | | 105.6 ± 7 | | 66 ± 6.6 |  |  |  |  |  |
|  |  |  |  |  |  | Sedentary | | | | | | | |  |  |  |  |  |
|  |  |  |  |  |  | 29.8 ± 3.4 | 65.2 ± 6.3 kg | | | 101.9 ± 8.4 | | 64.8 ± 4.5 | |  |  |  |  |  |
| Purdy, 2019, Canada | Uncomplicated | 37 | 17 | 20 | TM1, TM2, TM3 | TM1 | | | | | | | | 28 ± 6 | 28.8 ± 3.4 | | 112 ± 8 | 74 ± 7 |
|  |  |  |  |  |  | 31 ± 4 | | 23.4 ± 2.2 | | | 113 ± 10 | | 69 ± 7 |  |  |  |  |  |
|  |  |  |  |  |  | TM2 | | | | | | | |  |  |  |  |  |
|  |  |  |  |  |  | 31 ± 4 | | 23.0 ± 2.8 | | | 107 ± 9 | | 66 ± 7 |  |  |  |  |  |
|  |  |  |  |  |  | TM3 | | | | | | | |  |  |  |  |  |
|  |  |  |  |  |  | 32 ± 4 | | 23.1 ± 2.6 | | | 108 ± 5 | | 68 ± 7 |  |  | |  |  |
| van Doorn, 1992, USA | Uncomplicated | 33 | 33 | N/A | 16 | 30.9 ± 0.7 | 86 ± 1.7 kg | | | 110 ± 1.8 | | 73 ± 1.2 | | NA | NA | | NA | NA |
| **Crossover Trials** | | | | | | | | | | | | | | | | | | |
| Petrov Fieril, 2016, Sweden | Uncomplicated | 20 | 20 | N/A | 21 | Aerobic | | | | | | | | NA | NA | | NA | NA |
|  |  |  |  |  |  | 32.9 ± 4.3 | 20.0 ± 1.8 | | | 104 ± 13 | | 67 ± 6 | |  |  |  |  |  |
|  |  |  |  |  |  | Resistance | | | | | | | |  |  |  |  |  |
|  |  |  |  |  |  | 32.9 ± 4.3 | 20.0 ± 1.8 | | | 105 ± 8 | | 66 ± 4 | |  |  |  |  |  |
| de Oliveria, 2014, Brazil | Uncomplicated | 8 | 8 | 8 | 12-20 | NR | NR | | | 102 ± 6 | | 59.8 ± 6 | | NR | NR | | 102 ± 10 | 57 ± 10 |
| **Cohort** | | | | | | | | | | | | | | | | | | |
| Bisson, 2014, Canada | Uncomplicated | 61 | 61 | N/A | 12-15 | 30 ± 4.5 | 23.4 ± 4.2 | | | 102.9 ± 9.4 | | 65.1 ± 8.4 | | NA | NA | | NA | NA |
| Pivnarik, 1993, USA | Uncomplicated | 16 | 10 | 6 | 24-26 | 29 ± 5 | 65.1 ± 8.9 | | | SBP & DBP = NR  MAP = 84 ± 7 | | | | 29 ± 4 | 65.4 ± 4.4 | | NR | MAP = 85 ± 7 |
| Rafla, 1999, UK  Table 7. Characteristics of acute studies included in the systematic review | Uncomplicated | 143 | 143 | N/A | 36-40 | 25.5 | 66 kg | | | 112 | | 67 | | NA | NA | | NA | NA |
| Rafla, 2000, UK  Table 7. Characteristics of acute studies included in the systematic review | At risk (PIH) | 17 | 17 | N/A | 26-40 | A26 | NR | | | 149 | | 102 | | NA | NA | | NA | NA |
| Rauramo, 1988, Finland  Table 7. Characteristics of acute studies included in the systematic review | At risk (PE (n=13), DM (n=10), Cholestasis (n=8)) | 50 | 31 | 19 | 32-40 | PE | | | | | | | | 26 ± 3 | 70 ± 12 | | 112 ± 8 | 78 ± 7 |
|  |  |  |  |  |  | 27 ± 4 | 71 ± 8 | | | 140 ± 6 | | 98 ± 5 | |  |  |  |  |  |
|  |  |  |  |  |  | DM | | | | | | | |  |  |  |  |  |
|  |  |  |  |  |  | 27 ± 5 | 72 ± 9 | | | 118 ± 10 | | 77 ± 8 | |  |  |  |  |  |
|  |  |  |  |  |  | Chol | | | | | | | |  |  |  |  |  |
|  |  |  |  |  |  | 28 ± 4 | 74 ± 13 | | | 116 ± 9 | | 76 ± 7 | |  |  |  |  |  |
| Rauramo, 1988, Finland | Uncomplicated | 25 | 25 | NA | 32-38 | 26 ± 3 | 60 ± 12 | | | 112 ± 8 | | NR | | NA | NA | | NA | NA |
| Sady, 1990, USA | Uncomplicated | 45 | 45 | NA | 20-34 | 22 -37 | 69.9 ± 11.19 | | | NR | | NR | | NA | NA | | NA | NA |
| Santos, 2016, Brazil | Uncomplicated | 28 | 28 | NA | 30.51±3.3 | 26 ± 6.9 | 23.7 ± 3.2 | | | SBP & DB = NR  MAP = 81.4 ± 9.6 | | | | NA | NA | | NA | NA |

*PIH* pregnancy induced hypertension, *PE* preeclampsia, *DM* diabetes mellitus, *GDM* gestational diabetes mellitus, *NR* not reported, *TM1/2/3* trimester 1/2/3, *Con* control, *Int* intervention, *GA* gestational ag

Table S8. Design of Intervention Exercise Studies

| **Author** | **Mode** | **Frequency**  **(times/week)** | **Duration**  **(min)** | **Intensity** | **Length**  **(weeks)** | **Supervision** | **Control** |
| --- | --- | --- | --- | --- | --- | --- | --- |
| Bahadoran et al. 2015 | Walking | 3-5 | 30-45 | LMPA | 20 | Unsupervised | Routine care |
| Barakat et al. 2011 | Walking, core, stretches & aerobic dance | 3 | 35-45 | LMPA | 29-33 | Supervised | Not specified |
| Barakat et al. 2012 | Walking, core, stretches & aerobic dance | 3 | 40-45 | LMPA | 29-33 | Supervised | Not specified |
| Barakat et al. 2014 | Aerobic dance & resistance ex. | 3 | 55-60 | LMPA | 27-31 | Supervised | No exercise |
| Boparai et al. 2021 | Treadmill/cycle ergometer/elliptical | 3-4 | 25-40 | MPA | 15-19 | Partially supervised | Routine care |
| Brislane et al. 2021 | Cycle ergometer | 3-4 | 15-30 | MPA | 26 | Partially supervised | Routine care |
| Carpenter et al. 2015 | Recumbent cycle ergometer | 1 | 30-45 | LMPA | 20 | Supervised | Continued usual PA habits |
| Carpenter et al. 2017 | Aerobic & resistance ex. + pelvic floor | 1 | 20 | MPA | 20 | Supervised | Continued usual PA habits |
| Daniel et al. 2015 | Low impact aerobic dance | 2-3 | 45-60 | MPA | 8 | Supervised | Continued usual PA habits |
| de Oliveria et al. 2012 | Walking | 3 | 15 + | MPA | 18-25 | Supervised | Routine care |
| Erkkola et al. 1976 | All types | 3 | 60 | MVPA | 24-28 | Unsupervised | Routine care |
| Fernández-Buhigas et al. 2020 | Aerobic & resistance ex. + pelvic floor & balance | 3 | 60 | LMPA | 23-27 | Supervised | Continued usual PA habits |
| Ferriera et al. 2014 | PFMT | 1 | 35 | LMPA | 18 | Supervised | No control |
| Garnaes et al. 2016 | Treadmill walking & resistance ex. | 3 | 60 | VPA | 16-25 | Supervised | Continued usual PA habits |
| Guelfi et al. 2016 | Cycle ergometer | 3 | 20-60 | MVPA | 14 | Supervised | Routine care |
| Haakstad et al. 2016 | Aerobic dance & resistance ex. | 1 | 60 | MPA | 12 | Supervised | Continued usual PA habits |
| Halse et al. 2015 | Cycle ergometer | 3-5 | 25-45 | MVPA | 5-7 | Supervised | Routine care |
| Huifen et al. 2022 | Resistance ex. | 3 | 50-60 | MPA | >6 | Supervised | Routine care |
| Kasawara et al. 2013 | Cycle ergometer | 1 | 30 | LPA | 20-28 | Supervised | Routine care |
| Khoram et al. 2019 | Walking | 4 | 20-30 | MPA | 20 | Unsupervised | Routine care |
| Nascimento et al. 2011 | Resistance ex. | 1-5 | 40 | LMPA | 16-26 | Supervised + unsupervised | Routine care |
| O'Connor et al. 2011 | Resistance ex. | 2 | 45 | LMPA | 12 | Supervised | No control |
| Perales et al. 2016 | Aerobic & resistance ex. | 3 | 55-60 | LMPA | 30 | Supervised | Routine care |
| Petrov Fieril et al. 2015 | Resistance ex. | 2 | 60 | MVPA | 12 | Supervised | Education on exercises in pregnancy |
| Ramirez-Velez et al. 2011 | Aerobic & power ex. | 3 | 60 | MVPA | 16 | Supervised | Routine care |
| Rodríguez-Díaz et al. 2017 | Pilates | 2 | 40-45 | Not specified | 8 | Supervised | Routine care |
| Seneviratne et al. 2015 | Cycle ergometer | 3-5 | 15-30 | MPA | 16 | Unsupervised | No exercise |
| Silva-Jose et al. 2021 | Aerobic & resistance ex. + pelvic floor & balance | 3 | 55-60 | MPA | 30 | Virtually supervised | Routine care |
| Stutzman et al. 2010 | Walking | 3-5 | 0.6km/day working up to 3km/day | LPA | 16 | Unsupervised | Continued usual PA habits |
| Vladimirov et al. 2015 | Aerobic – Medical Pole Walking | 7 | 25-30 | MPA | 3 | Supervised | Walking/gymnastics stretching + routine care |
| Yeo et al. 2000 | Treadmill & cycle ergometer | 3 | 45 | MPA | 10 | Supervised | Continued usual PA habits |
| Yeo et al. 2008 | Walking | 5 | 40 | MPA | 18-22 | Unsupervised | Stretching exercises |

*PA* physical activity, *LPA* light physical activity, *LMPA* light to moderate physical activity, *MPA* moderate physical activity, *MVPA* moderate to vigorous physical activity, *VPA* vigorous physical activity, *ex.* exercise/s, *PFMT* pelvic floor muscle training, *km* Kilometers

Table S9. Design of Acute Exercise Studies

| **Author** | **Mode** | **Duration (min)** | **Intensity** | **Control** |
| --- | --- | --- | --- | --- |
| Amorim et al. 2018 | Treadmill or cycle ergometer | 20 | MPA | No control |
| Avery et al. 1999 | Resistance exercise | 60 | LMPA | Non-pregnant control |
| Babbar et al. 2016 | Supervised Yoga | 60 | LPA | Non exercising - PPT presentation |
| Bgeginski et al. 2015 | Resistance exercise | 40 | 50% 1RM | Non-pregnant control |
| Bisson et al. 2014 | Treadmill | >10 | To volitional fatigue | No control |
| Brun et al. 2011 | Resistance exercises in bed | 30 | LPA | No exercise, listening to music |
| de Oliveria et al. 2014 | Cycle ergometer | 30 | LMPA | Non exercising |
| Jeffreys et al. 2006 | Resistance ex. (supine) | 10 | MVPA | No control |
| Kim et al. 2018 | Resistance ex. in bed | 30 | LPA | Non exercising |
| Meah et al. 2021 | Cycle ergometer | To 70% HRR | MVPA | Non-pregnant control |
| Meah et al. 2021 | Resistance ex. | Not specified | MPA | Non-pregnant control |
| Morrow et al. 1989 | Cycler ergometer | 5 | LMPA | No control |
| O'Neill et al. 2006 | Cycle ergometer upright vs semi-rec | 12 | MPA | No control |
| O'Neill et al. 1993 | Treadmill | 26 | LMPA | No control |
| Petrov Fieril et al. 2016 | Nordic walking or resistance exercise | 30 | LMPA | No control |
| Pijpers et al. 1984 | Semi-rec cycle ergometer | 20 | LMPA | Non exercising |
| Pivnarik et al. 1993 | Cycle ergometer | 15 | MVPA | Physically active vs sedentary |
| Purdy et al. 2019 | Peak cycle ergometer Test | To volitional fatigue | To volitional fatigue | Non-pregnant control |
| Rafla et al. 1999 | Cycle ergometer | 5 | LMPA | No control |
| Rafla et al. 2000 | Cycle ergometer | 5 | LMPA | No control |
| Rauramo et al. 1988 | Cycle ergometer | 5 | MPA | Healthy pregnant women |
| Rauramo et al. 1988 | Cycle ergometer | 6 | VPA | No control |
| Sady et al. 1990 | Cycle ergometer | To volitional fatigue | To volitional fatigue | No control |
| Santos et al. 2016 | Ramp treadmill test | 11.41 ± 4.23 | To volitional fatigue | No control |
| Sklempe Kokic et al. 2018 | Treadmill | 20 | MVPA | No control |
| van Doorn et al. 1992 | Max cycle ergometer Test | To volitional fatigue | To volitional fatigue | No control |
| Webb et al. 1994 | Submaximal cycle ergometer test | 15 | MVPA | Non exercising |

*PA* physical activity, *LPA* light physical activity, *LMPA* light to moderate physical activity, *MPA* moderate physical activity, *MVPA* moderate to vigorous physical activity, *VPA* vigorous physical activity, *ex.* exercise/s, *PFMT* pelvic floor muscle training, *RM* repetition maximum, *Semi-rec* semi-recumbent, *HRR* heart rate reserve
